# TGF-β Regulates DNA Methyltransferase Expression in Prostate Cancer, Correlates with Aggressive Capabilities, and Predicts Disease Recurrence

**DOI:** 10.1371/journal.pone.0025168

**Published:** 2011-09-30

**Authors:** Qiang Zhang, Lin Chen, Brian T. Helfand, Thomas L. Jang, Vidit Sharma, James Kozlowski, Timothy Michael Kuzel, Lihua J. Zhu, Ximing J. Yang, Borko Javonovic, Yinglu Guo, Scott Lonning, Jay Harper, Beverly A. Teicher, Charles Brendler, Nengwang Yu, William J. Catalona, Chung Lee

**Affiliations:** 1 Department of Urology, Northwestern University Feinberg School of Medicine, Chicago, Illinois, United States of America; 2 Robert H. Lurie Comprehensive Cancer Center, Northwestern University, Chicago, Illinois, United States of America; 3 The Cancer Institute of New Jersey, Robert Wood Johnson Medical School/University of Medicine and Dentistry of New Jersey, New Brunswick, New Jersey, United States of America; 4 Department of Medicine, Northwestern University Feinberg School of Medicine, Chicago, Illinois, United States of America; 5 Program in Gene Function and Expression, University of Massachusetts Medical School, Boston, Massachusetts, United States of America; 6 Department of Pathology, Northwestern University Feinberg School of Medicine, Chicago, Illinois, United States of America; 7 Department of Preventive Medicine, Northwestern University Feinberg School of Medicine, Chicago, Illinois, United States of America; 8 Institute of Urology, Department of Urology, The First Hospital, Peking University, Beijing, China; 9 The Genzyme Corporation, Framingham, Massachusetts, United States of America; 10 Department of Surgery, Northshore University Heathsystem, Evanston, Illinois, United States of America; University of Hong Kong, Hong Kong

## Abstract

**Background:**

DNA methyltransferase (DNMT) is one of the major factors mediating the methylation of cancer related genes such as TGF-β receptors (TβRs). This in turn may result in a loss of sensitivity to physiologic levels of TGF-β in aggressive prostate cancer (CaP). The specific mechanisms of DNMT's role in CaP remain undetermined. In this study, we describe the mechanism of TGF-β-mediated DNMT in CaP and its association with clinical outcomes following radical prostatectomy.

**Methodology/Principal Findings:**

We used human CaP cell lines with varying degrees of invasive capability to describe how TGF-β mediates the expression of DNMT in CaP, and its effects on methylation status of TGF-β receptors and the invasive capability of CaP in vitro and in vivo. Furthermore, we determined the association between DNMT expression and clinical outcome after radical prostatectomy. We found that more aggressive CaP cells had significantly higher TGF-β levels, increased expression of DNMT, but reduced TβRs when compared to benign prostate cells and less aggressive prostate cancer cells. Blockade of TGF-β signaling or ERK activation (p-ERK) was associated with a dramatic decrease in the expression of DNMT, which results in a coincident increase in the expression of TβRs. Blockade of either TGF-β signaling or DNMT dramatically decreased the invasive capabilities of CaP. Inhibition of TGF-β in an TRAMP-C2 CaP model in C57BL/6 mice using 1D11 was associated with downregulation of DNMTs and p-ERK and impairment in tumor growth. Finally, independent of Gleason grade, increased DNMT1 expression was associated with biochemical recurrence following surgical treatment for prostate cancer.

**Conclusions and Significance:**

Our findings demonstrate that CaP derived TGF-β may induce the expression of DNMTs in CaP which is associated with methylation of its receptors and the aggressive potential of CaP. In addition, DNMTs is an independent predictor for disease recurrence after prostatectomy, and may have clinical implications for CaP prognostication and therapy.

## Introduction

TGF-β is a pleiotropic growth factor that has been implicated in multiple, and often diametrically opposed functions, including cell proliferation, cell growth arrest, differentiation, and apoptosis [1], [2]. An obvious question raised by these diverse functions is how TGF-β mediates these seemingly contradictory roles in both cancer and benign cells. In cancer cells, TGF-β acts as a growth promoter and aids in metastasis, whereas in normal cells it appears to inhibit cell growth and induce apoptosis [3]. Characteristics of aggressive prostate cancer (CaP) include a gradual loss of sensitivity to TGF-β and over-expression of TGF-β, which appears to initiate a vicious cycle for tumor progression. Although it is well known that a reduction or loss of expression of TGF-β receptors (TβRs) enables cancer cells to escape the growth inhibitory effect of TGF-β and to gain a growth advantage, the cellular mechanism(s) underlying these events in human CaP cells remains undefined. Previously, we have demonstrated that the loss of TβRs expression by promoter methylation is associated with insensitivity to TGF-β-mediated growth inhibition [4].

DNA methylation is carried out by DNA methyltransferases (DNMTs). There are at least three functional DNMTs that have been identified in eukaryotic systems. DNMT1 has been implicated primarily in the maintenance of methylation patterns that occurs during cellular replication, and it preferentially methylates hemi-methylated DNA [5]. It has been the most extensively studied maintenance methyltransferase and is abundant in tumor cells and tissues. In comparison, DNMT2 does not appear to have significant methylation activity and DNMT3L is likely to be limited to DNA methylation during germline development [5]. Finally, DNMT3A and DNMT3B are known to be de novo methylators of CpG sites [6], which have higher methyltransferase activity for unmethylated DNA than DNMT1 and can contribute to de novo methylation during embryogenesis [7], [8]. Although DNMT is reported to be associated with some aggressive cancers like hepatocellular carcinomas, stomach cancers, non-small cell lung cancers, lymphoma and prostate cancers [9], [10], [11], [12], [13], its role remains controversial and the overall regulation, coordination and activity of DNMTs is unclear with different cancers. Furthermore, the mechanism of DNMTs in cancer cells and its association with invasive malignant capabilities and clinical outcomes after treatment have not been described.

We recently reported that the epigenetic regulation of TGF-β-induced expression of Foxp3 may be mediated through the inactivation of extracellular signal-regulated kinases (ERK), which may down-regulate DNMTs in benign cells [14]. As stated above, CaP cells and tissue are insensitive to TGF-β-mediated growth inhibition and have promoter methylation patterns which decrease the expression of TβRs (TβRI and TβRII) [4], [15], [16]. Taken together, these results indicate that the insensitivity to TGF-β in some CaP cells is at least partly due to the promoter methylation of TβRs. These findings have led us to explore the following two hypotheses in the present study: 1) There may be crosstalk between tumor derived TGF-β and DNMTs which is related to methylation in cancer; 2) DNMTs may be closely associated with the prostate cancer progression and outcomes following radical prostatectomy. To our knowledge, this subject matter has yet to be reported.

The purpose of our study was several-fold. First, we sought to investigate the corresponding changes in DNMT and TβRs expression and ERK activation after treating CaP cells with varying degrees of invasive capability and benign prostate epithelial cells with TGF-β. Next, we examined the effect of a neutralizing TGF-β antibody on the expression of DNMTs and tumor growth in vivo using a xenograft model. Finally, we determined whether activation of DNMTs was associated with biochemical recurrence following radical prostatectomy.

## Materials and Methods

(A detailed explanation is presented in Method S1)

### Cell Lines

The mouse CaP cell line TRAMP-C2 cells was obtained from Dr. N. Greenberg [12]. The benign human prostate epithelial cell line, RWPE-1, was purchased from American type culture collection (ATCC). BPH-1 cells were kindly provided by Dr. Simon Hayward. Four variants of the human CaP PC-3 cell lines (PC-3, PC-3M-Pro4, PC-3M and PC-3M-LN4) with varying degrees of invasive capabilities were kindly provided by Dr. Fidler and Dr. Pettaway [17], [18], [19], [20]. The reason we chose PC-3 variants was because these variants originate from the same cell line, but vary in their aggressive capabilities. The results of signaling regulation were more comparable in contrast to using the different kinds of CaP cell lines. For some experiments, cells were rendered insensitive to TGF-β (as a negative control) by introducing a TβRIIDN as previously described [21], [22]. In some experiments, cells were treated with or without TGF-β1 or MEK inhibitor U0126 (Promega). Finally, some experiments involved the use of Anti-TGF-β (1, -2, -3) neutralizing mAb (clone 1D11; a gift from Genzyme Corporation) as previously described [23], [24]. (Method S1).

### TGF-β1 ELISA

RWPE-1, BPH-1 and all PC3 variants and the corresponding TβRIIDN infected cell lines were cultured in fresh serum-free media for 24 hours. TGF-β1 ELISA was carried out using the Human TGF-β1 Immunoassay Kit (R&D Systems) (Method S1).

### [^3^H]-Thymidine Incorporation Assay

All cells were grown in culture for 48 hours. Cells were then exposed to a medium containing [^3^H]-thymidine (0.5 µCi/mL; Amersham Biosciences) for an additional 5 hours. Thymidine incorporation was expressed as the fraction of counts found in cells of untreated controls (Method S1).

### Western blot analysis

Western blot analyses were performed to compare TβRs, DNMTs and ERK expression after different treatments over time (Method S1).

### Methylation-Specific PCR (MSP) and Sequencing

MSP for the methylation status of TβRs was performed according to our previous report [4]. The methylated sites in cytosine positions with/without treatment of 5-Aza or TGF-β were identified.

### Immunofluorescence and Co-staining

Immunofluorescence studies were performed on all PC-3 cell line derivatives as previously described [22], [25]. For co-localization of DNMTs and phosphorylated ERK (p-ERK), cells were analyzed by using nucleus (DAPI)-DNMTs(TR)-p-ERK (FITC) triple staining (Method S1).

### Quantitative RT-PCR

Human benign prostate epithelial cells RWPE-1 and BPH-1, and CaP PC-3 serials) were cultured in fresh media for 24 hours, then exposed for 24 hours to either: 1) external recombinant TGF-β1 (10 ng/ml), 2) anti-TGF-β neutralizing monoclonal Ab (1D11; 5 µg/ml), or 3) MEK inhibitor U0126 (5 µM). Total RNA was extracted using an RNeasy kit (Qiagen). Primers for human for DNMTs [26] and TβRs [4] were listed in Method S1.

### Cell invasion assay

Cell invasion assay (Matrigel invasion assay) was done in a 24-well Transwell chamber (8 µm pore size; CytoSelect; Cell Biolabs). Cells were plated at a density of 0.5×10^6^ to 1.0×10^6^/mL in serum-free medium. TGF-β1 and/or Erk inhibitor UO126, 5-Aza were added directly to the cell suspension, and 24 h later, the suspension was aspirated and the invaded cells were counted with a light microscope under high magnification objective (×100; Olympus) and measured at A560 nm in a plate reader after treatment with the extraction solution.

### Animal Studies

The study was initiated using the subcutaneous (sc) injection of mouse prostate cancer TRAMP-C2 cells transfected with HSV1-tk-GFP-luciferase (SFG-nTGL) reporter gene expression vector [27], [28] into the right flank region of 30 C57BL/6 mice as described earlier [25]. Animals were randomly assigned to one of three groups following intraperitoneal injections with the specific anti-TGF-β neutralizing antibody 1D11 or control antibody 13C4 as described before [23], [24]. All the mice were sacrificed after 15 injections of antibodies and group 3 were sacrificed on the same time interval. (Method S1). This study received approval from the institutional review board of Northwestern University (Evanston, IL). Northwestern University ACUC Approval protocol number 2007-0565.” (Letter S1).

### Construction of Tissue Microarrays (TMAs) and Clinical Outcome Assement

The existing clinical case information and banked tissue established within our prostate SPORE program database at Northwestern University was used. All enrolled subjects provided written informed consent by Northwestern Memorial Hospital and the study was approved by the Northwestern University Institutional Review Board (The IRB number is 1480-002, Letter S2). A total of 243 radical prostatectomy specimens were available with associated clinical information. A series of prostate TMAs were constructed with formalin-fixed, paraffin-embedded radical prostatectomy specimens as described previously [4], (Method S1 and Method S2).

### Immunohistochemistry

All antibodies raised against DNMTs, phosphorylated ERK (p-ERK), total ERK (t-ERK), phosphorylated Smad2, TβRI and TβRII were first tested and optimized on whole-tissue sections and test arrays as previously described [4], [17], [29], [30], (Method S1 and Method S2).

***Statistical Analysis.*** The SPSS 10.0.7 software package (SPSS, Inc.) was used for all analyses. Kaplan-Meier survival curve was analyzed by the log-rank test using the Graphpad Prism 4.02 software (Graphpad Software) (Method S1).

## Results

### 1. DNMTs expression is associated with down regulation of TβRs and more invasive prostate cancer phenotypes

**Figure 1:**
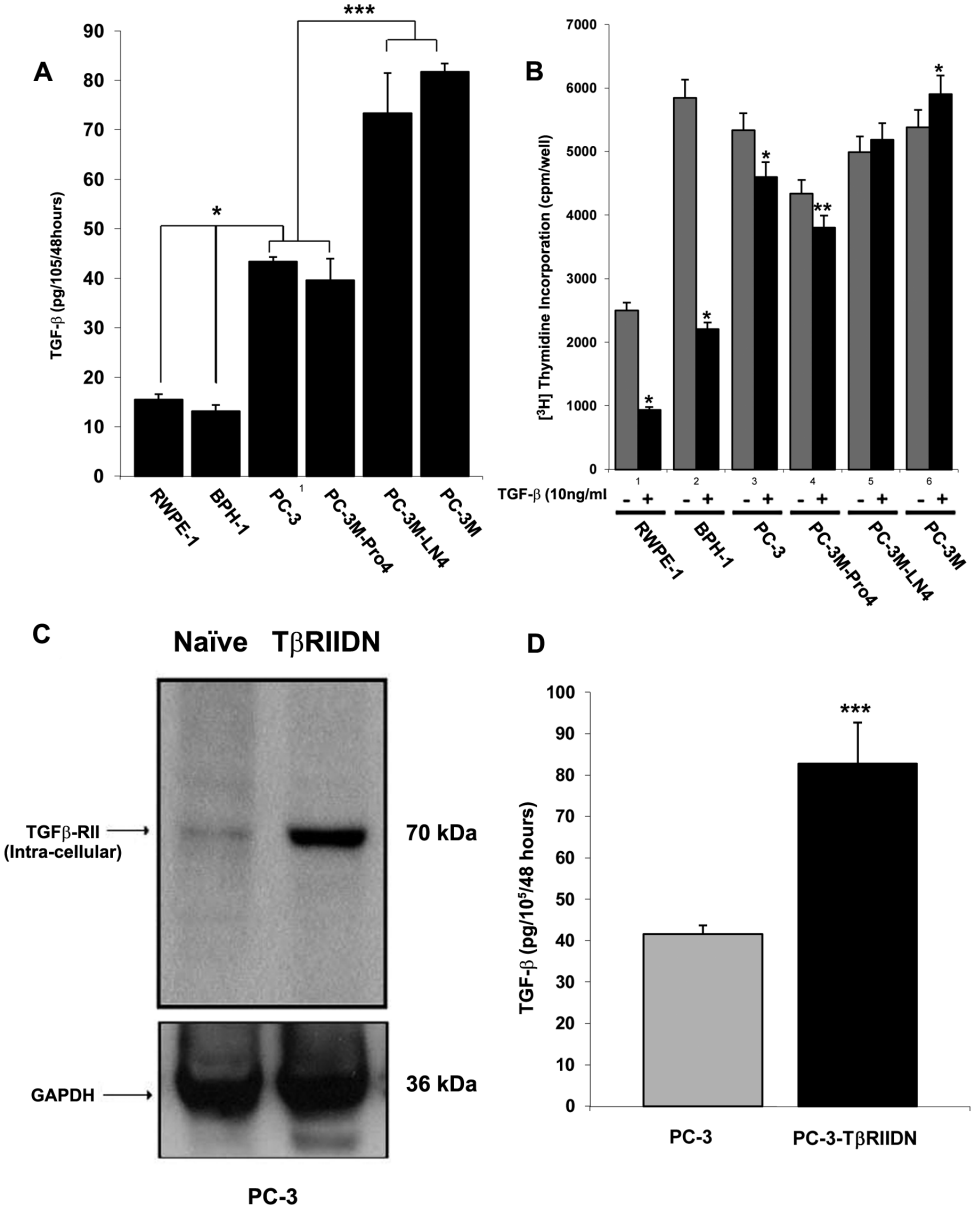
Tumor derived TGF-β regulates the expression of TβRs and secretion of TGF-β. **A.** ELISA assay demonstrating that PC-3 cell lines express significantly (p<0.05) higher (×2–6 times) levels of TGF-β compared to benign prostate cell lines, BPH-1 and RWPE-1. Furthermore, PC-3M and PC-3M-LN4, secreted almost 2 times higher baseline levels of TGF-β1 compared to PC-3 and PC-3M-Pro4 cells, respectively, which are less invasive. **B.** A thymidine incorporation assay indicates that the growth of RWPE-1 and BPH-1 cells is inhibited significantly by exposure to TGF-β1. In comparison, the growth of PC-3 and PC-3M-Pro4 cells is only slightly inhibited, and PC-3M-LN4 and PC-3M cells show no significant response to TGF-β1 exposure. **C.** In all cancer cell lines (here we show PC-3 as an example), inhibition of TGF-β using the TβRIIDN construct results in significantly higher naïve TβRII expression, and **D.** higher TGF-β secretion. Similar findings are found in the findings in the more invasive cell lines. In comparison, there was no difference in the expression of TβRII in BPH-1 or RWPE-1 when they were infected with TßRIIDN (Table S1).

An ELISA assay was initially performed to determine whether there were differences in the endogenous expression levels of TGF-β in different CaP cell lines when compared to benign prostate cell lines. We found that all PC-3 cell lines expressed significantly higher levels of TGF-β (×2 to 6 times) compared to the BPH-1 and RWPE-1 (p<0.05). Furthermore, we found that more invasive cells (PC-3M and PC-3M-LN4) secreted almost 2 times higher baseline levels of TGF-β1 when compared with the less invasive cell lines (PC-3 and PC-3M-Pro4) (Fig. 1A).

We confirmed that different prostate cell lines behave differently in response to exogenous TGF-β1 exposure. For example, we found that RWPE-1 and BPH-1 cells were most sensitive to exogenous TGF-β1 as their growth was inhibited by 64.1% and 61.9%, respectively, after 24 hours of treatment with TGF-β1. In comparison, PC-3 and PC-3M-Pro4 cells were only inhibited by 13.7% and 12.3%, respectively. Finally, the growth rate of PC-3M-LN4 and PC-3M was unaffected by TGF-β1 exposure (Fig. 1B). Interestingly, in CaP cell lines, inhibition of TGF-β signaling, using the dominant negative type II TGF-β receptor (TßRIIDN) construct, was associated with significantly higher endogenous TβRII expression (using antibodies directed against the intracellular domain, because TβRIIDN includes only extracellular and transmembrane domains, but not intracellular domain) (Fig. 1C) and higher TGF-β secretion (Fig. 1D). In comparison, there was no difference in the expression of TGF-β in BPH-1 or RWPE-1 when they were infected with the retroviral TβRIIDN construct (Table S1).

**Figure 2:**
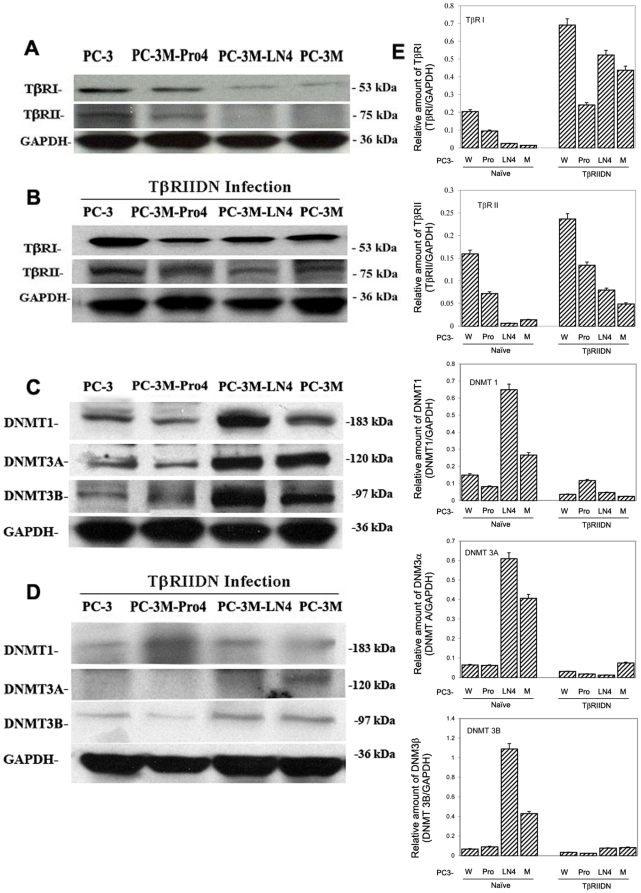
TGF-β induced expression of DNMTs is inversely associated with the expression of TβRs. **A.** Western blot analyses demonstrate that in contrast to the expression of TGF-β, both TβRI and TβRII expression (as described in Figure 1B) is significantly reduced in the more invasive cell lines compared with less invasive cell lines. **B.** Blockade of TGF-β signaling with the TβRIIDN causes significant increase in the expression TβRIs in all cell lines. **C.** In contrast to the expression of TβRs, the over expression of DNMTs is associated with more invasive cell lines compared with the less invasive cell lines. **D.** Blockade of TGF-β signaling with TβRIIDN caused a ≥3-fold decrease in the expression of DNMTs. The corresponding value (relative ratio of TβRs/GAPDH, or DNMTs/GAPDH) is shown in right charts. (Fig. 2A and 2B were from the same Western Blot image, and Fig. 2C and 2D were from the other single Western blot image).

In contrast to the expression of TGF-β, both TβRI and TβRII expression was significantly reduced in the more invasive cell lines, PC-3M-LN04 and PC-3M, compared with PC-3 and PC-3M-Pro4 cells (Fig. 2A). Blockade of TGF-β signaling with the TβRIIDN vector caused an approximately two to ten-fold increase in the expression of both TβRI and TβRII in all CaP cell lines (Fig. 2B). Taken together this suggests that increased baseline levels of TGF-β are associated with the inhibition of TβRs expression. Blockade of intracellular TGF-β signaling resulted in up-regulation of secretion of TGF-β in cancer cells.

Since promoter methylation of TβRs is associated with decreased expression [4], we compared the expression levels of DNMTs in the different CaP cell lines. In general, the more invasive PC-3M-LN4 and PC-3M cells showed an increased expression of DNMTs, when compared to the less invasive PC-3 and PC-3M-Pro4 (Fig. 2C). Blockade of TGF-β signaling with the TβRIIDN vector caused a ≥3-fold decrease in the expression of DNMTs in all CaP cell lines (Fig. 2D), and there was a corresponding increase in the expression of both TβRI and TβRII (Fig. 2B). The corresponding value (relative ratio of TβRs/GAPDH, or DNMTs/GAPDH) is shown in right panels. This finding was also supported by additional confirmatory studies. Immunoblot analyses demonstrated that after treatment with 5-Aza-2′-deoxycytidine (5-Aza), the expression of TβRI and TβRII in PC-3 increased dramatically. In contrast, the expression of both TβRI and TβRII decreased significantly with the treatment of TGF-β and this change could be recovered when 5-Aza is added (Figure S1A). Similarly, real-time PCR confirmed that the expression of both TβRI and TβRII was increased 2 to 2.5 folds after treatment of 5-Aza in PC-3 cells. Treatment with TGF-β suppressed the expressions of TβRI and TβRII 46% and 29% respectively (Figure S1B). We also identified the methylation status of TβRI and TβRII promoters, by using the same MSP approach and sequencing methodologies [4]. Using this technique, we found the same methylated sites as our previous study [4] in that cytosine positions −251, −231, −244, −348, −356 and −365 in the promoter of TβRI, and +27, +32 and −140 for the promoter of TβRII were methylated (Figure S1C). PC-3 cells also have a portion of TβRI and TβRII promoters that are unmethylated. Interestingly, treatment with TGF-β increased the methylation status, but treatment with 5-Aza converted all methylated sites to unmethylated. The thymidine incorporation assay indicated that the proliferation of PC-3 cells were only modestly inhibited modestly by exogenous TGF-β. In comparison, 5-Aza treatment resulted in significant inhibition of cell proliferation, regardless of whether exogenous TGF-β was added into the culture or not. There was no significant difference observed between treatment with both 5-Aza and TGF-β or with 5-Aza alone (P>0.05) (Figure S1D).

### 2. DNMTs expression is mediated through a phosphorylated-ERK dependent pathway

**Figure 3:**
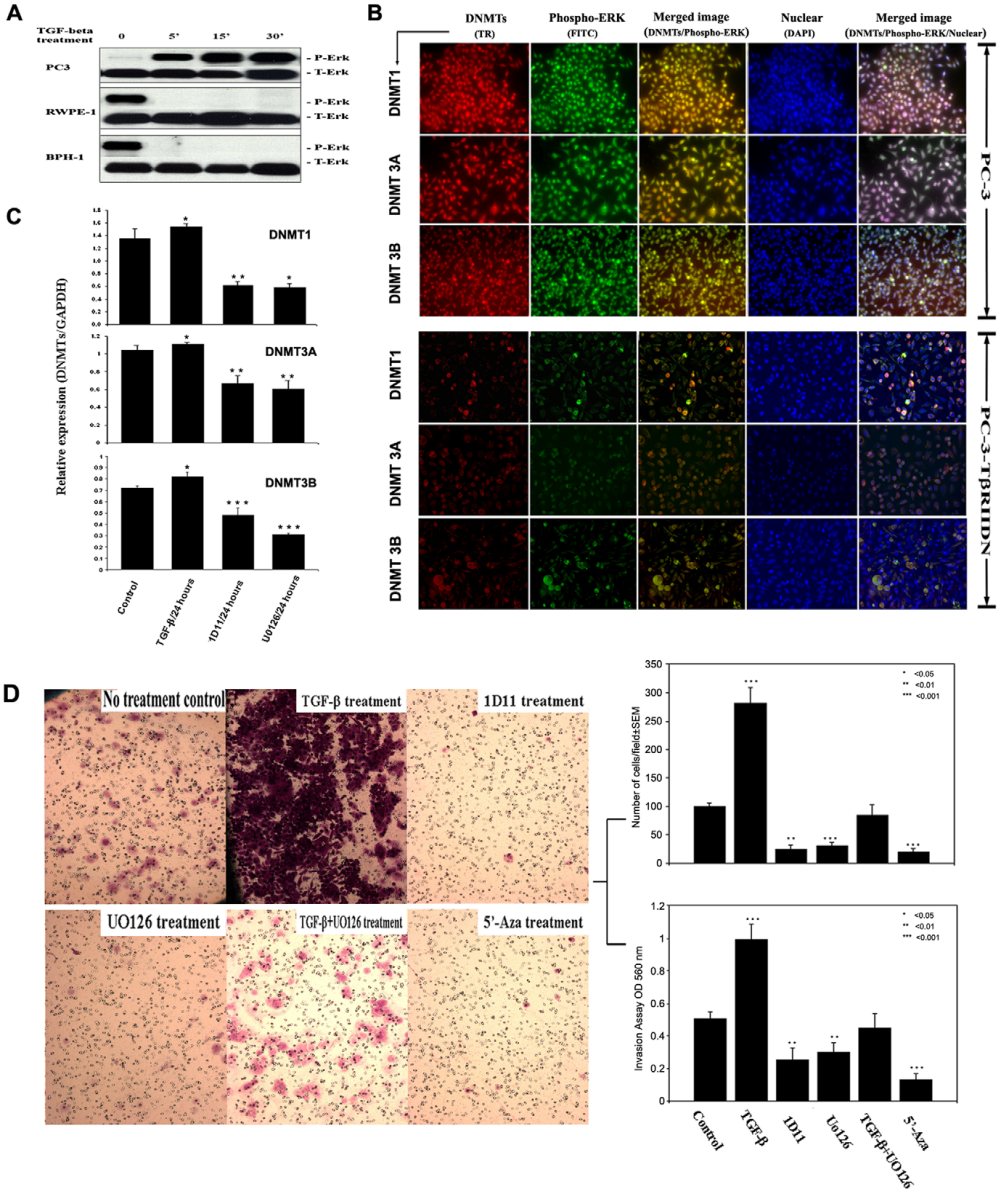
TGF-β induced DNMTs is mediated by ERK activation. **A.** The benign BPH-1 and RPWE-1 cells express significantly higher baseline levels of p-ERK than the PC-3 cells. There is a time dependent positive correlation between treatment with TGF-β1 and the expression of p-ERK in PC-3 cells. The levels of p-ERK continue to increase during all subsequent time points up to 30 minutes after TGF-β1 addition. In contrast, the expression of p-ERK is rapidly (<5 minutes) inhibited after TGF-β1 exposure in benign cells in a fashion that is independent of the total ERK protein expression. **B.** Immunofluorescence reveals that only cells (this is PC3 for example) expressing p-ERK exhibit DNMT expression. In contrast, when PC-3 cells are rendered insensitive to TGF-ß1 by TβRIIDN, levels of both p-ERK and DNMT are significantly reduced (magnification: 10×20). **C.** We performed real time PCR to better quantify the relationship between TGF-β1, p-ERK and DNMTs. Exposure to TGF-β1 significantly increased the expression of all three DNMTs in PC-3 cells. Treatment with 1D11, or MEK inhibitor, UO126 is associated with the down-regulation of all DNMT mRNA expression. **D.** (Here we showed most aggressive PC-3M as a sample). There was a significant increase in cell motility through a Matrigel-coated polycarbonate membrane under the treatment of TGF-β1 (10 ng/mL). The invasion of all CaP cells could be inhibited by blocking the TGF-β signal by 1D11 or using a p-ERK inhibitor UO126, or DNMT inhibitor 5-Aza separately. The inhibition of invasion by UO126 can't be reverted by TGF-β treatment. Upper right panel: Corresponding numbers of invasive cells. Bottom right panel: absorbance values. This result indicates p-ERK mediated TGF-β-induced DNMT potentiates the invasive ability of prostate cancer cell lines. (magnification, 10×10).

Our previous studies demonstrate that ERK may influence DNMT expression in benign cells [14]. We therefore sought to determine whether the level of activated ERK (phosphorylated ERK; p-ERK) is related to TGF-β-induced expression of DNMTs. To test this hypothesis, we first determined the level of p-ERK in benign prostate cells and compared it to the levels in different CaP cell lines. BPH-1 and RPWE-1 cells expressed significantly higher baseline levels of p-ERK than PC-3 cells (Fig. 3A). Interestingly, the time course of p-ERK expression after exposure to TGF-β was different between the benign and malignant cell lines. Specifically, there was a time dependent positive correlation between treatment with TGF-β1 and the expression of p-ERK in all PC-3 cell lines. In fact, this rapid increase in p-ERK expression (4-fold) began within 5 minutes following TGF-β1 treatment. The levels of p-ERK continued to increase during all subsequent time points up to 30 minutes after TGF-β1 addition. In contrast, the expression of p-ERK was rapidly inhibited (<5 minutes) after TGF-β1 addition to the media of benign cells, in a fashion that was independent of the total ERK protein expression (Fig. 3A). Immunofluorescence studies were subsequently used to help determine whether p-ERK and DNMTs were co-localized to the same cellular regions. To this end, confocal microscopic analyses of formaldehyde fixed immunostained PC-3 cells, in the absence or presence of TGF-β1, demonstrated co-loczalization between p-ERK and DNMTs signals. Only the cells with p-ERK immunofluorescence exhibited DNMT expression. In contrast, when PC-3 cells were rendered insensitive to TGF-β1 by transfection with the TβRIIDN, levels of both p-ERK and DNMTs were reduced dramatically as determined by immunofluorsence staining (Fig. 3B). To better quantify this relationship between TGF-β1, p-ERK and DNMTs, we next used real time PCR. These results demonstrated that exposure to TGF-β1 for 24 hours significantly increased the expression of all three DNMTs (∼16.7%–14%) in all PC-3 cell lines studied. Treatment with an antibody specific for TGF-β1 (1D11; 5 mg/ml) or the specific ERK inhibitor, UO126, led to significant down-regulation of DNMTs mRNA expression (∼33.9%–52.3%, and ∼41.5%–57.6% respectively, Fig. 3C). These results suggest that TGF-β mediated expression of DNMTs is associated with an increase in p-ERK in cancer cells. Specifically, tumor derived TGF-β appears to be responsible for this ERK activation, as blockade of the original secreted TGF-β resulted in a great change in the expression of DNMTs (Fig. 3C). These results also suggest that tumor derived TGF-β mediated ERK activation is at least one of the major mediators for TGF-β induced expression of DNMTs which lead to TβRs down-regulation by promoter methylation in CaP [4], [14]. After treatment with TGF-β, there was a significant increase in the invasive capabilities of CaP cells. Invasion of CaP cells was inhibited by either TGF-β inhibitor 1D11, or p-Erk inhibitor U0126 or DNMT inhibor 5-Aza. The inhibition of invasion by the U0126 could not be reversed by TGF-β1 treatment. Importantly, DNMTs inhibitor 5-Aza can dramatically inhibited the CaP cells invasion, even more than blockade of TGF-β or p-ERK (Fig. 3D). This observation suggested that p-ERK was downstream factor of TGF-β, and synergistically mediates TGF-β regulated DNMTs which was closely associated with the invasive capability of CaP cells.

### 3. In vivo validation of the effects of TGF-β on ERK activation, DNMT expression, and prostate cancer growth

**Figure 4:**
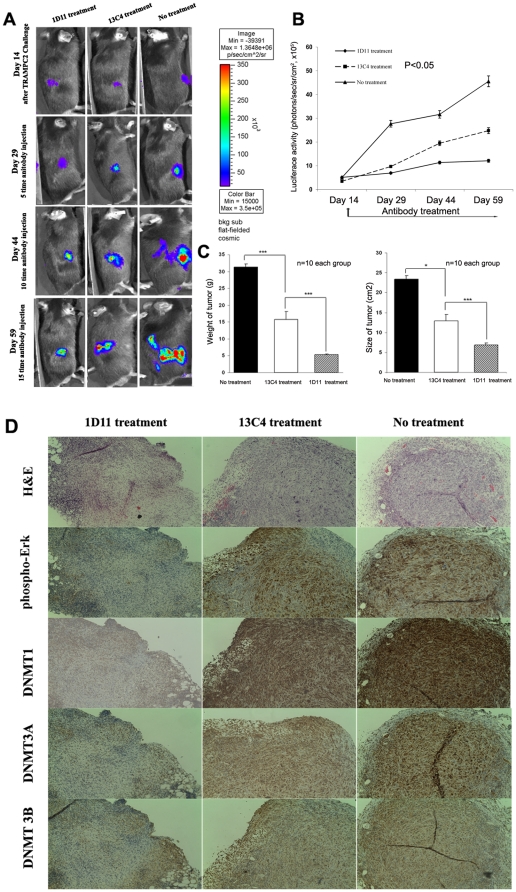
TGF-β induced DNMTs is associated with growth of prostate cancer in vivo. **A.** IVIS 100 imaging system was used to monitor tumor growth in real-time. We found that tumor growth is inhibited dramatically with the treatment of 1D11 compared with Group 2 (13C4 treatment) and 3 (No treatment control). **B.** 1D11 treatment inhibits the tumor growth in a time dependent manner. **C,** At the end of the 45-day treatment period, mice were sacrificed and tumors were isolated. The average tumor weight and volume was 5.3 g and 6.85 cm^3^, respectively in 1D11 treatment group. In comparison, the average weight and volume of tumors in the 10 animals treated with the control 13C4 was significantly greater at 15.8 g and 12.85 cm^3^, respectively. The corresponding values in the mice that received no treatment were 31.4 g and 23.39 cm^3^, respectively (*P*<0.01 among three groups). **D.** Immunohistochemical analyses of the primary tumors revealed that the expression of p-ERK, DNMTs in animals with 1D11 treatment is significantly lower than those of the other two groups.

To validate whether TGF-β is responsible for the activation of ERK and up-regulation of DNMTs which may be involved in tumor progression in vivo, we conducted experiments using a mouse xenograft CaP model which involved the injection of CaP tumor cells (TRAMP-C2 cells stably transfected with a HSV1-tk-GFP-luciferase reporter, 5×10^6^/each mouse). Tumor growth was followed using luciferase imaging. We used three groups of mice to better understand the effects of TGF-β on ERK activation and DNMT expression: Group 1: mice (n = 10) received regular injections of the TGF-β neutralizing antibody, 1D11. Group 2: mice (n = 10) received the isotype control antibody, 13C4, at the same regular intervals as Group 1. Group 3: received no treatment after xenograft injection as a control. We found that tumor growth was significantly inhibited with anti-TGF-β 1D11 antibody, treatment (Group 1) compared with the other two groups (Fig. 4A, 4B). In fact, at the end of the 45-day treatment period, one of the ten mice (10%) in this group was free of tumor. In the remaining 9 mice, the average tumor weight and volume was 5.3 g and 6.85 cm^3^, respectively. In comparison, tumors were found in all mice in Groups 2 and 3. The average weight and volume of tumors in the 10 animals treated with the control antibody (Group 2) or no treatment (Group 3) was significantly greater (Fig. 4C). There were no metastases in all the groups as assessed by bioluminescence imaging. Immunohistochemical analyses of the primary tumors revealed that the expression of p-ERK and DNMTs in animals in Group 1 were significantly lower than those of the other two groups (Fig. 4D).

### 4. DNMTs correlates with clinical characteristics

**Figure 5:**
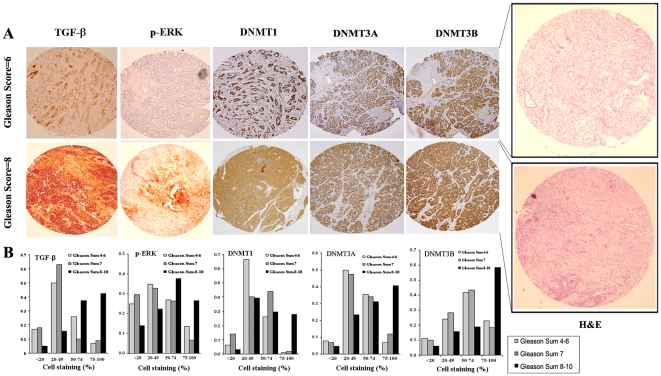
Confirmation of p-ERK mediated, TGF-β-induced DNMT in TMA specimens obtained at the time of prostatectomy. **A.** Immunohistochemical analysis of serial TMA sections from a patient with Gleason's score of 8, revealed higher expression of TGF-β, p-ERK, DNMTs, but lower expression of TβRI and TβRII and p-Smad2 as compared to serial sections taken from a patient with a lower Gleason's grade of 6. These results are representative of the predominant staining pattern seen in all patient samples/tissue arrays (magnification: 10×20). The corresponding frequency (or percentage) of staining and intensity of staining could be found on B. **B.** High levels of TGF-β1 expression (Score = 3) were identified in 42.3%, 8.9% and 7.0%, p-Erk expression in 36.4%, 6.7% and 13.5%, DNMT1 expression in 27.9%, 1.8% and 1.3%, DNMT3A expression in 40.6%, 11.9% and 6.7%, and DNMT3B expression in 58.7%, 18.3% and 22.8% of high (≥8), intermediate ( = 7), and low Gleason grade (≤6), respectively.

To evaluate the association between TGF-β and the induction of DNMTs in CaP specimens, we compared the expression levels of TGF-β1, ERK, p-ERK, TβRI, TβRII, p-Smad2, and DNMTs in archived tissue microarray specimens obtained at the time of radical prostatectomy and correlated them with corresponding patients' clinical and pathologic characteristics (Table S2. Each marker was assigned a value of 0 (<20% cell immunostaining), 1 (20–49% cell immunostaining), 2 (50–74% cell immunostaining) and 3 (75–100% cell immunostaining) depending on the percentage of cancer cells showing positive immunostaining. The positive and negative control staining was showed in the “Figure S2”. We found that a high level of expression of TGF-β1, p-ERK and DNMTs coupled with a low level of expression of TβRI, TβRII, and p-Smad2 was associated with adverse pathologic features, such as higher Gleason's grade (Fig. 5A, Fig. 5B, Table S3). These results correspond to our finding in PC-3M-LN4 and PC-3M cells that TGF-β induced DNMTs are associated with clinically more aggressive phenotypes.

We found a significant correlation between the expression of TGF-β1 and DNMTs in these tissue microarray specimens. There was also a significant correlation between TGF-β and p-ERK, TGF-β and TβRI, p-ERK and DNMT1, p-ERK and DNMT3A, p-ERK and DNMT3B respectively. In addition, we found a significant correlation between the expression levels of all three of the DNMTs. There were inverse relationships between DNMTs and TβRs, DNMT1.vs. TβRI, DNMT1.vs. TβRII, DNMT3A vs. TβRII (Table S3).

### 5. DNMTs is associated with biochemical recurrence in prostate cancer patients after radical prostatectomy

To examine the utility of these markers as possible prognostic tools, we correlated the expression levels of the above TGF-β related biomarkers of each tumor with the clinical outcome of the corresponding patient using the database of Northwestern University's Prostate SPORE. The log rank test was used determine whether or not these various markers correlated with biochemical recurrence (PSA>0.2 ng/ml after radical prostatectomy). Variables of interest included all TMA markers, clinical stage, clinical Gleason's score, which was grouped as 4–6, 7, 8–10, surgical margin status, PSA doubling time, and patient age. As mentioned above, all specimens were assigned a value between 0–3 based upon the percentage of cancer cells showing a positive staining. A Kaplan Meier curve was generated for each of the above significant variables.

**Figure 6:**
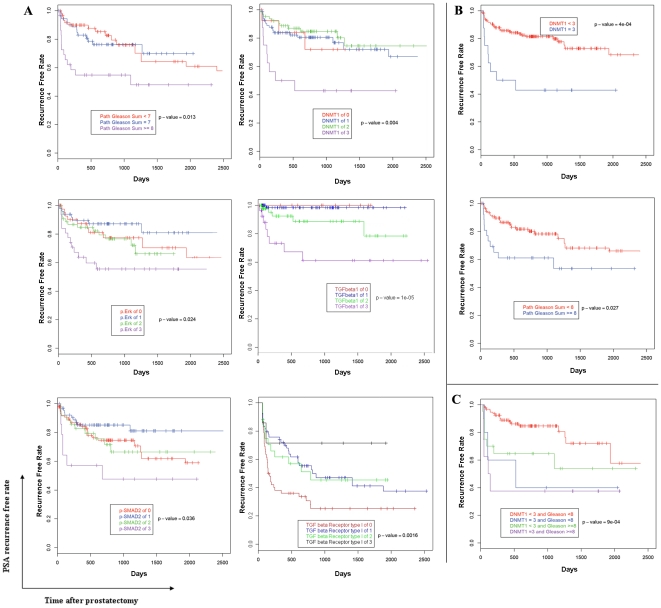
TGF-β-induced DNMT1 predicts prostate cancer recurrence. **A.** Kaplan Meier curve was generated for significant variables. TGF-β1, p-Smad2, p-ERK, pathologic Gleason Score and DNMT1, TβRI were all predictors of biochemical recurrence. **B.** DNMT1, pathologic Gleason score was analyzed as two different groups, and there was a significant difference in the survival curves. **C.** Cox Proportional Hazards Model only includes DNMT1, grouped as below 3 and 3, and pathologic Gleason score sum of patients, grouped as below 8, or above. Using our dataset, patients with tissue level DNMT1 of 3 had a 3.53 times higher biochemical recurrence rate than patients with lower tissue levels of DNMT1. Patients with Gleason score sum ≥8 have a 2.27 times higher biochemical recurrence rate compared to patients with Gleason score sum <8.

Expression levels of TGF-β1, p-Smad2, p-ERK, pathologic Gleason Score and DNMT1, TβRI were associated with biochemical recurrence after radical prostatectomy (Fig. 6A. B, Table S4). The degree of DNMT1 expression correlated significantly with biochemical recurrence [P = 0.0043 using 4 expression groups; (0, 1, 2, 3; P = 4×10^−04^ using 2 groups; low expression (0, 1, 2) vs high expression(3)]. DNMT3A and DNMT3B, surgical margin status, TGF-β type II receptor expression level and PSA doubling time were not associated with biochemical recurrence (p>0.05). To determine the best model for predicting PSA recurrence, a Cox Proportional Hazards Model was fit to include all the significant variables and backward selection method was used to eliminate non-significant variables. The final selected model includes DNMT1, grouped as below 3 (low expression) or above 3 (high expression; log Rank P = 0.002; hazard ratio = 3. 53; 95% CI 1.6–7.78), and pathologic Gleason score sum of patients, grouped as below 8, or above (log Rank P = 0.034; hazard ratio = 2.27; 95% CI 1. 06–4.83) (Fig. 6C, Table S5). Patients whose tumors had a DNMT1 expression level of 3 (high expression) had a 3.53 higher risk of recurrence than patients with lower scores of DNMT1 in the tumor. Even in patients with low Gleason grade (≤6), there was a high risk of recurrence if high levels of DNMT1 expression were present. A high DNMT1 expression was independently associated with biochemical recurrence, irrespective of Gleason score. There was no correlation between PSA doubling time and the expression levels of DNMT1.

## Discussion

Many malignancies, including CaP, exhibit aberrant methylation within the promoter regions of genes associated with a loss of function [31], [32], [33]. Presumably, this loss of function contributes to the development and progression of the disease. DNMTs are the major mediators responsible for the hypermethylation of the promoter regions of many genes encoding for signaling factors including the TβRs promoter [4], which may subsequently inhibits TβRs translation which ultimately results in the insensitivity to the normal inhibitory effects of TGF-β, uninhibited growth and progression of cancer [4], [34], [35], [36]. Although DNMTs are recognized as important regulators of transcription of carcinogenesis [37], [38], [39], [40], [41], [42], and have been a topic of considerable interest in the last few years, their assessment in vivo and within human specimens remains uncertain. Our study findings demonstrate that high level of expression of DNMTs is associated with more aggressive phenotypes of CaP, lower expression of TβRs, and lower sensitivity to the inhibitory role of TGF-β.

The molecular mechanisms which govern regulation of DNMTs have been largely unknown [43], and the relationship(s) between DNMTs and TGF-β in CaP have yet to be explored. Although other factors like c-Jun may be involved in the process [44], ERK appears to be an obligatory switch for TGF-β-mediated expression of DNMTs in CaP, although the effect of TGF-β on ERK activation remains controversial [45], [46]. More recently we reported that there was a differential activation of ERK between benign and malignant cells in response to TGF-β [14], [47]. In our prior studies involving benign cells, we reported that TGF-β exposure, ERK inactivation and DNMTs down regulation contribute to the expression of Foxp3 in benign immune cells [14]. In the present study, higher expression levels of DNMTs were found to be associated with CaP with higher invasive capabilities when compared with CaP cells with lower invasive capabilities. Interestingly, we found that increased levels of DNMTs were associated with increased levels of TGF-β and p-ERK, and decreased levels of TβRs. In contrast, our hypotheses were verified by a serial of blockade assays, blockade of TGF-β signaling using the TβRIIDN or neutralizing antibody 1D11, decreased the levels of DNMTs between 50%–90% in more invasive cell lines, and to a lesser degree (30–50%) in the less invasive cell lines. These findings indicate that tumor-derived TGF-β is a major mediator involved in the regulation of DNMTs and TβRs in human CaP cells, and this process correlates with more invasive phenotypes. Meanwhile, down regulation of DNMT expression by blockade of TGF-β is associated with an up-regulation of naïve TβRs expression. These findings, taken together with results from our previous study, suggest that tumor-derived TGF-β activates ERK, which mediates the expression of DNMTs (because blockade of ERK resulted in 50% decrease on DNMTs expression). DNMTs then methylate the TGF-β receptor promoter regions resulting in the loss of growth inhibition mechanisms which we reported earlier [4]. Our present study also provides insight into the interaction between ERK and DNMTs in CaP. Exposure to the ERK inhibitor, UO126 results in >50% reduction in the expression of DNMTs, indicating that ERK is one of the major regulators of TGF-β induced DNMTs expression in CaP cells. Our observations of the co-localization of p-ERK and DNMTs also suggest that only cells which exhibit ERK activation can express DNMTs, which is evidence that they are in the same TGF-β activated signal pathway.

Importantly, we found direct evidence that blockade of DNMT by its inhibitor 5-Aza resulted in decrease in the invasive capabilities of CaP, as well as the blockade of either TGF-β by 1D11, or blockade of p-ERK by UO126. This data indicates that DNMT is a major promoter for CaP invasive capabilities. This procedure is regulated by TGF-β and mediated by p-ERK.

Based upon the above findings, we postulate that tumor-derived TGF-β can regulate its receptors by a potential feedback loop which is mediated by activation of ERK. Some other signaling factors like Serine/threonine protein phosphatases 2 (PP2A) [48] may be involved in this procedure. P-ERK may subsequently activate the transcription factors in the DNMTs promoter and increases the expression of DNMTs which methylates TGF-β receptor promoter regions resulting in the loss of growth inhibition mechanisms that are normally induced by TGF-β. Simultaneously, the downregulation of TβR expression and low level of TGF-β signaling may act as a positive feedback mechanism to induce the reflexive stimulation of TGF-β secretion in CaP. These potential feedback loops could explain the reduced expression of TβRs and large amounts of TGF-β that have been observed in advanced CaP. Our in vivo xenograft model also demonstrated that inhibition of DNMTs correlated to a lower tumor weight and cancer proliferation rate. These results suggest that the expression of DNMTs is associated with aggressive malignant phenotypes, tumor growth, and progression in vivo. In combination with our previous findings [4], we found that DNMTs is an important factor and predictor related to CaP progression.

Furthermore, the close correlation between TGF-β, ERK and DNMTs in tissue microarray specimens indicates that this cascade of signal events is likely not only associated with aggressive malignant phenotypes in vitro, but may also be involved with progression of CaP in humans. Based on our results, during progression of prostate cancer, an attenuation of expression of TGF-β receptors facilitates tumor cells escaping from the growth inhibition by TGF-β which is Smad dependent. Meanwhile, the Smad-independent pathway, such as p-ERK and DNMT signaling could be induced by TGF-β and results in the more aggressive phenotypes.

Our data shows that increased expression of DNMTs is highly correlated with both the expression levels of TGF-β1 and p-ERK. Furthermore, there was a significant correlation between the levels of DNMTs and Gleason grade, which further supports our findings that DNMTs are associated with more invasive CaP phenotypes. This finding is similar to recent reports suggesting that DNMT1 is associated with lung cancer progression [49]. The present results demonstrate that DNMT1 is associated with biochemical recurrence in CaP patients seven years following radical prostatectomy. Thus, patients with higher tissue expression levels of DNMT1 are at increased risk for biochemical recurrence compared to those with lower tissue expression levels. The relationship between DNMT1 expression and biochemical recurrence is independent of Gleason grade. Although other variables including TGF-β1, p-ERK, Gleason grade were also showed significantly associated with biochemical recurrence, the final Cox Proportional Hazards Model demonstrated that DNMT1, in combination with pathologic Gleason sum, are stronger predictors for disease outcome. The exact mechanism of this observation remains unclear, but variables involved in the signal pathway including tumor expression of DNMT1, TGF-β1, and p-ERK may be useful in predicting clinical outcome following radical prostatectomy. High expression level of DNMT1 was risk factors for biochemical recurrence in men with CaP, regardless of Gleason's score.

In summary, our findings indicate that DNMTs expression levels are correlated with invasive capabilities in cultured human CaP cell lines. Additionally, we found that tumor-derived TGF-β and ERK are involved in the regulation of DNMTs in these cell lines. Inhibition of TGF-β in vivo results in the corresponding inhibition of DNMTs, and appears to significantly decrease tumor growth. In addition, we confirmed that the expression levels of TGF-β, ERK and DNMTs in tissue specimens obtained at the time of prostatectomy mimicked our findings in cell culture. Finally, we found that high expression levels of DNMT1 may potentially be used to predict biochemical recurrence in patients following radical prostatectomy.

## Supporting Information

Figure S1 **Immunoblot analyses demonstrated that after treatment with 5-Aza-2′-deoxycytidine (5-Aza), the expression of TβRI and TβRII in PC-3 increased dramatically.** In contrast, the expression of both TβRI and TβRII decreased significantly with the treatment of TGF-β and this change could be recovered when 5-Aza is added (Figure S1A). Similarly, real-time PCR confirmed that the expression of both TβRI and TβRII was increased 2–2.5 folds after treatment of 5-Aza in PC-3 cells. Treatment of TGF-β suppressed the expressions of TβRI and TβRII 46% and 29% respectively (Figure S1B). We also identified the methylation status of TβRI and TβRII promoters, by using the same MSP approach and sequencing methodologies (4). Using this technique, we found the same methylated sites as our previous study (4) in that cytosine positions −251, −231, −244, −348, −356 and −365 in the promoter of TβRI, and +27, +32 and −140 for the promoter of TβRII were methylated (Figure S1C). PC-3 cells also have a portion of TβRI and TβRII promoters that are unmethylated. Interestingly, treatment with TGF-β increased the methylation status, but treatment with 5-Aza converted all methylated sites to unmethylated. The thymidine incorporation assay indicated that the proliferation of PC-3 cells were only modestly inhibited modestly by exogenous TGF-β. In comparison, 5-Aza treatment resulted in a significant inhibition of cell proliferation, regardless of whether exogenous TGF-β was added into the culture or not. There was no significant difference observed between treatment with both 5-Aza and TGF-β or with 5-Aza alone (P>0.05) (Figure S1D). Taken together, these results support our above finding that knockdown of DNA methyltransferases result in the demethylation of the TGF-β receptors gene promoters and restoration of TGF-beta inhibition of cell growth. TGF-β contributes to the methylation of its own receptors. **A.** Immunoblot analyses demonstrated that after treatment with 5-Aza-2′-deoxycytidine (5-Aza), the expression of TGF-β receptor I (TβRI) and TGF-β receptor II (TβRII) in PC-3 increased dramatically. In contrast, the expression of both TβRI and TβRII decreased significantly with the treatment of TGF-β and this change could be recovered when 5-Aza is added. **B.** Real-time PCR confirmed that the expression of both TβRI and TβRII was increased 2–2.5 folds after treatment of 5-Aza in PC-3 cells. Treatment of TGF-β suppressed the expressions of TβRI and TβRII 46% and 29% respectively. **C.** The methylation status of TβRI and TβRII promoters was identified by using the same MSP approach and sequencing methodologies (4). The methylated sites in cytosine positions −251, −231, −244, −348, −356 and −365 in the promoter of TβRI, and +27, +32 and −140 for the promoter of TβRII. PC-3 also has portion of TβRI and TβRII promoters which are unmethylated. Interestingly, treatment with TGF-β also increased the methylation status, but treatment with 5-Aza can convert all methylated sites to unmethylated. **D.** The thymidine incorporation assay indicated that the proliferation of PC-3 could be inhibited little by exogenous TGF-β. However, 5-Aza treatment resulted in a significant inhibition of cell proliferation, regardless of whether exogenous TGF-β was added into the culture or not. There was no significant difference observed between treatment with both 5-Aza and TGF-β or with 5-Aza alone (P>0.05).
(TIF)

Figure S2 **For positive control staining for TMA staining, a tissue (Colon cancer for TGF-β, Breast cancer for p-ERK, Placenta for DNMT1 and DNMT3A, Breast cancer for DNMT3B respectively) which is well known to have expression of target protein was used.** Negative controls were identical array sections stained in the absence of primary antibody.
(TIF)

Method S1 **Supplemental Materials and Methods.**
(DOC)

Method S2 **NU Pathology Core Facility Standard Operating Procedure.**
(DOC)

Table S1 **Secretion of TGF-β in various derivatives of PC3 cells and benign cells (pg/ml/48 hours/10^5^ cells).**
(DOC)

Table S2 **Clinical and pathologic characteristics of patients who experienced a radical prostatectomy.**
(DOC)

Table S3 **Correlation of TGF-β signaling components and clinical characterics.**
(DOC)

Table S4 **The variables significantly correlated with biochemical recurrence.**
(DOC)

Table S5 **The significant variables selected by Cox Proportional Hazards Mode.**
(DOC)

Letter S1 **Northwestern University ACUC Approval protocol number 2007-0565.**
(PDF)

Letter S2 **Approval letter by the Northwestern University Institutional Review Board (The IRB number is 1480-002).**
(PDF)

## References

[1] Sintich SM, Lamm MLG, Sensibar Jam Lee C (1999). Transforming growth factor-ß1 induced proliferation of the prostate cancer cell line, TSU-Pr1: the role of platelet-derived growth factor.. Endocrinology.

[2] Zhou W, Park I, Pins M, Kozlowski JM, Jovanovic B (2003). Dual regulation of proliferation and growth arrest in prostatic stromal cells by transforming growth factor-ß1.. Endocrinology.

[3] Pardali K, Moustakas A (2007). Actions of TGF-beta as tumor suppressor and pro-metastatic factor in human cancer.. Biochim Biophys Acta.

[4] Zhang Q, Rubenstein JN, Jang TL, Pins M, Javonovic B (2005). Insensitivity to transforming growth factor-beta results from promoter methylation of cognate receptors in human prostate cancer cells (LNCaP).. Mol Endocrinol.

[5] Robertson KD (2001). DNA methylation, methyltransferases and cancer.. Oncogene.

[6] Bestor TH (2000). DNA-methyltransferase of mammals.. Hum Mol Genet.

[7] Okano M, Bell DW, Haber DA, Li E (1999). Dnmt3a and Dnmt3b are essential for de novo methylation and mammalian development.. Cell.

[8] Lu R, Wang X, Chen ZF, Sun DF, Tian XQ (2007). Inhibition of the extracellular signal-regulated kinase/mitogen-activated protein kinase pathway decreases DNA methylation in colon cancer cells.. J Biol Chem.

[9] Saito Y, Yamazoe T, Qin Z, Ohgomori K, Mochitate K (2003). Increased protein expression of DNA methyltransferase (DNMT) 1 is significantly correlated with the malignant potential and poor prognosis of human hepatocellular carcinomas.. Int J Cancer.

[10] Etoh T, Kanai Y, Ushijima S, Nakagawa T, Nakanishi Y (2004). Increased DNA methyltransferase 1 (DNMT1) protein expression correlates significantly with poorer tumor differentiation and frequent DNA hypermethylation of multiple CpG islands in gastric cancers.. Am J Pathol.

[11] Xing J, Stewart DJ, Gu J, Lu C, Spitz MR (2008). Expression of methylation-related genes is associated with overall survival in patients with non-small cell lung cancer.. Br J Cancer.

[12] Amara K, Ziadi S, Hachana M, Soltani N, Korbi S (2010). DNA methyltransferase DNMT3b protein overexpression as a prognostic factor in patients with diffuse large B-cell lymphomas.. Cancer Sci.

[13] Chen MF, Chen WC, Chang YJ, Wu CF, Wu CT (2010). Role of DNA methyltransferase 1 in hormone-resistant prostate cancer.. J Mol Med.

[14] Luo X, Zhang Q, Liu V, Xia Z, Pothoven KL (2008). Cutting edge: TGF-beta-induced expression of Foxp3 in T cells is mediated through inactivation of ERK.. J Immunol.

[15] Kim IY, Ahn HJ, Zelner DJ, Shaw JW, Lang S (1996). Loss of expression of transforming growth factor-β receptors type I and type II correlates with tumor grade in human prostate cancer tissues.. Clinical Cancer Research.

[16] Kim IY, Ahn HJ, Zelner DJ, Shaw JW, Sensibar JA (1996). Genetic change in transforming growth factor-β (TGF-β) receptor type I gene correlates with insensitivity to TGF-β1 in human prostate cancer cells.. Can Res.

[17] Pettaway CA, Pathak S, Greene G, Ramirez E, Wilson MR (1996). Selection of highly metastatic variants of different human prostatic carcinomas using orthotopic implantation in nude mice.. Clin Caner Res.

[18] Kozlowski JM, Fidler IJ, Nicholson G (1988). Prostate cancer and the invasive phenotype: application of new in vivo and in vitro approaches.. Tumor progression and metastasis.

[19] Kozlowski JM, Rosen S, Lee C, Tallman MS (1991). Growth requirements of human prostate cancers in vitro and in vivo.. Innovations in Urologic Oncology: Selected Papers from a Northwestern University Cancer Center Symposium.

[20] Lee C, Shevrin DH, Kozlowski JM (1993). In vivo and in vitro approaches to study metastasis in human prostatic cancer.. Cancer Met Rev.

[21] Zhang Q, Yang X, Pins M, Javonovic B, Kuzel T (2005). Adoptive transfer of tumor-reactive transforming growth factor-beta-insensitive CD8^+^ T cells: eradication of autologous mouse prostate cancer.. Cancer Res.

[22] Zhang Q, Helfand BT, Jang TL, Zhu LJ, Chen L (2009). Nuclear factor-kappaB-mediated transforming growth factor-beta-induced expression of vimentin is an independent predictor of biochemical recurrence after radical prostatectomy.. Clin Can Res.

[23] Liu VC, Wong LY, Jang T, Shah AH, Park I (2007). Tumor evasion of the immune system by converting CD4+CD25- T cells into CD4^+^CD25^+^ T regulatory cells: role of tumor-derived TGF-beta.. J Immunol.

[24] Perry K, Wong L, Liu V, Park I, Zhang Q (2008). Treatment of transforming growth factor-beta-insensitive mouse Renca tumor by transforming growth factor-beta elimination.. Urology.

[25] Zhang Q, Yang XJ, Kundu SD, Pins M, Javonovic B (2006). Blockade of transforming growth factor-{beta} signaling in tumor-reactive CD8(+) T cells activates the antitumor immune response cycle.. Mol Cancer Ther.

[26] Attwood J, Richardson B (2004). Relative quantitation of DNA methyltransferase mRNA by real-time RT-PCR assay.. Methods Mol Biol.

[27] Serganova I, Moroz E, Vider J, Gogiberidze G, Moroz M (2009). Multimodality imaging of TGFbeta signaling in breast cancer metastases.. FASEB J.

[28] Ponomarev V, Doubrovin M, Serganova I, Vider J, Shavrin A (2004). Novel triple-modality reporter gene for whole-body fluorescent, bioluminescent, and nuclear noninvasive imaging.. Eur J Nucl Med Mol Imaging.

[29] Steg A, Vickers SM, Eloubeidi M, Wang W, Eltoum IA (2007). Hedgehog pathway expression in heterogeneous pancreatic adenocarcinoma: implications for the molecular analysis of clinically available biopsies.. Diagn Mol Pathol.

[30] Grizzle WE, Myers RB, Manne U, Srivastava S, Hanausek M, Walaszek Z (1998). Immunohistochemical Evaluation of biomarkers in prostate and colorectal neoplasia: Principles and guidelines.. Methods in Molecular Medicine, Vol 14: Tumor Marker Protocols.

[31] Baylin SB, Herman JG, Graff JR, Vertino PM, Issa JP (1998). Alterations in DNA methylation: A fundamental aspect of neoplasia.. Adv Cancer Res.

[32] Chan MF, Liang G, Jones PA, Jones PA, Vogt PK (2000). Relationship between transcription and DNA methylation.. DNA methylation and cancer.

[33] Bender CM, Pao MM, Jones PA (1998). Inhibition of DNA methylation by 5-AZA-2′ deoxycytidine suppresses the growth of human tumor cell lines.. Cancer Res.

[34] Santi DV, Garrett CE, Barr PJ (1983). On the mechanism of inhibition of DNA-cytosine methyltransferases by cytosine analogs.. Cell.

[35] Wu JC, Santi DV (1987). Kinetic and catalytic mechanism of HhaI methyltransferase.. J Biol Chem.

[36] Bacolla A, Pradhan S, Roberts RJ, Wells RD (1999). Recombinant human DNA (cytosine-5) methyltransferase II Steady-state kinetics reveal allosteric activation by methylated DNA.. J Biol Chem.

[37] Deng C, Lu Q, Zhang Z, Rao T, Attwood J (2003). Hydralazine may induce autoimmunity by inhibiting extracellular signal-regulated kinase pathway signaling.. Arthritis Rheum.

[38] Lu Q, Wu A, Richardson BC (2005). Demethylation of the same promoter sequence increases CD70 expression in lupus T cells and T cells treated with lupus-inducing drugs.. J Immunol.

[39] Yu N, Wang M (2008). Anticancer drug discovery targeting DNA hypermethylation.. Curr Med Chem.

[40] Ghoshal K, Bai S (2007). DNA methyltransferases as targets for cancer therapy.. Drugs Today (Barc).

[41] Gowher H, Jeltsch A (2004). Mechanism of inhibition of DNA methyltransferases by cytidine analogs in cancer therapy.. Cancer Biol Ther.

[42] Fandy TE (2009). Development of DNA methyltransferase inhibitors for the treatment of neoplastic diseases.. Curr Med Chem.

[43] Patra SK, Patra A, Zhao H, Dahiya R (2002). DNA methyltransferase and demethylase in human prostate cancer.. Mol Carcinog.

[44] MacLeod AR, Rouleau J, Szyf M (1995). Regulation of DNA methylation by the Ras signaling pathway.. J Biol Chem.

[45] Lee MK, Pardoux C, Hall MC, Lee PS, Warburton D (2007). TGF-beta activates Erk MAP kinase signalling through direct phosphorylation of ShcA.. EMBO J.

[46] Giehl K, Seidel B, Gierschik P, Adler G, Menke A (2000). TGF beta1 represses proliferation of pancreatic carcinoma cells which correlates with Smad4-independent inhibition of ERK activation.. Oncogene.

[47] Lin C, Zhang Q, Helfand B, Qin W, Sharma V (2010). Erk Activation mediates Transforming Growth Factor-ß-induced up-regulation of DNA methyltransferase in human prostate cancer cells..

[48] Zhang Q, Chen L, Helfand B, Kozlowski J, Brendler C (2011). The recruitment of PP2A by TGF-β receptors mediates the response to TGF-β-induced activation of ERK in prostate cancer..

[49] Lin RK, Hsieh YS, Lin P, Hsu HS, Chen CY (2010). The tobacco-specific carcinogen NNK induces DNA methyltransferase 1 accumulation and tumor suppressor gene hypermethylation in mice and lung cancer patients.. J Clin Invest.

