# Supplementary figures and images for: TGF-β Regulates DNA Methyltransferase Expression in Prostate Cancer, Correlates with Aggressive Capabilities, and Predicts Disease Recurrence
Source: PLoS One. 2011 Sep 30;6(9):e25168. doi: 10.1371/journal.pone.0025168 (PMC3184137; doi:10.1371/journal.pone.0025168)

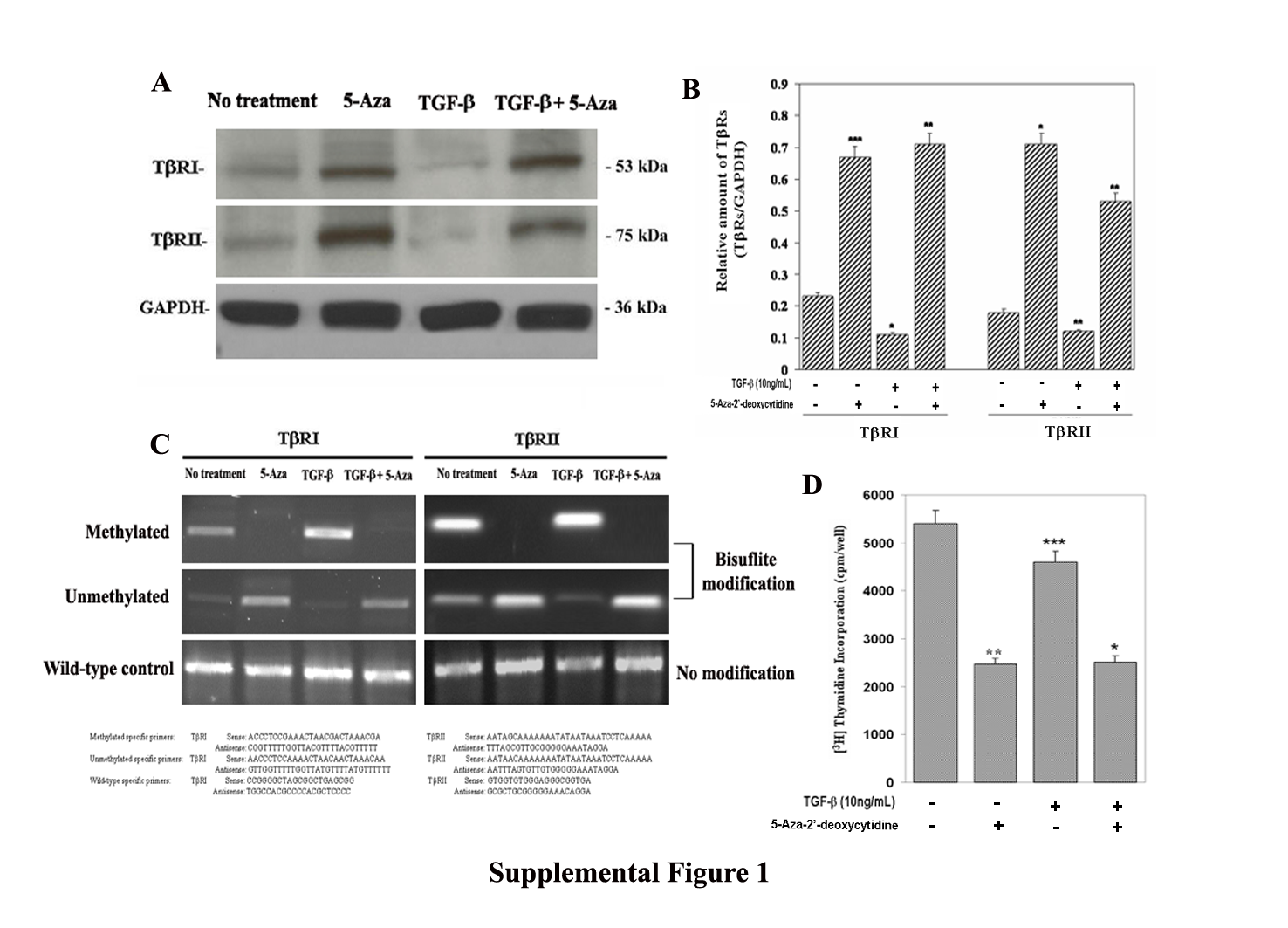

Supplement: Figure S1 — Immunoblot analyses demonstrated that after treatment with 5-Aza-2′-deoxycytidine (5-Aza), the expression of TβRI and TβRII in PC-3 increased dramatically. In contrast, the expression of both TβRI and TβRII decreased significantly with the treatment of TGF-β and this change could be recovered when 5-Aza is added (Figure S1A). Similarly, real-time PCR confirmed that the expression of both TβRI and TβRII was increased 2–2.5 folds after treatment of 5-Aza in PC-3 cells. Treatment of TGF-β suppressed the expressions of TβRI and TβRII 46% and 29% respectively (Figure S1B). We also identified the methylation status of TβRI and TβRII promoters, by using the same MSP approach and sequencing methodologies (4). Using this technique, we found the same methylated sites as our previous study (4) in that cytosine positions −251, −231, −244, −348, −356 and −365 in the promoter of TβRI, and +27, +32 and −140 for the promoter of TβRII were methylated (Figure S1C). PC-3 cells also have a portion of TβRI and TβRII promoters that are unmethylated. Interestingly, treatment with TGF-β increased the methylation status, but treatment with 5-Aza converted all methylated sites to unmethylated. The thymidine incorporation assay indicated that the proliferation of PC-3 cells were only modestly inhibited modestly by exogenous TGF-β. In comparison, 5-Aza treatment resulted in a significant inhibition of cell proliferation, regardless of whether exogenous TGF-β was added into the culture or not. There was no significant difference observed between treatment with both 5-Aza and TGF-β or with 5-Aza alone (P>0.05) (Figure S1D). Taken together, these results support our above finding that knockdown of DNA methyltransferases result in the demethylation of the TGF-β receptors gene promoters and restoration of TGF-beta inhibition of cell growth. TGF-β contributes to the methylation of its own receptors. A. Immunoblot analyses demonstrated that after treatment with 5-Aza-2′-deoxycytidine (5-Aza), the exp [file pone.0025168.s001.tif]

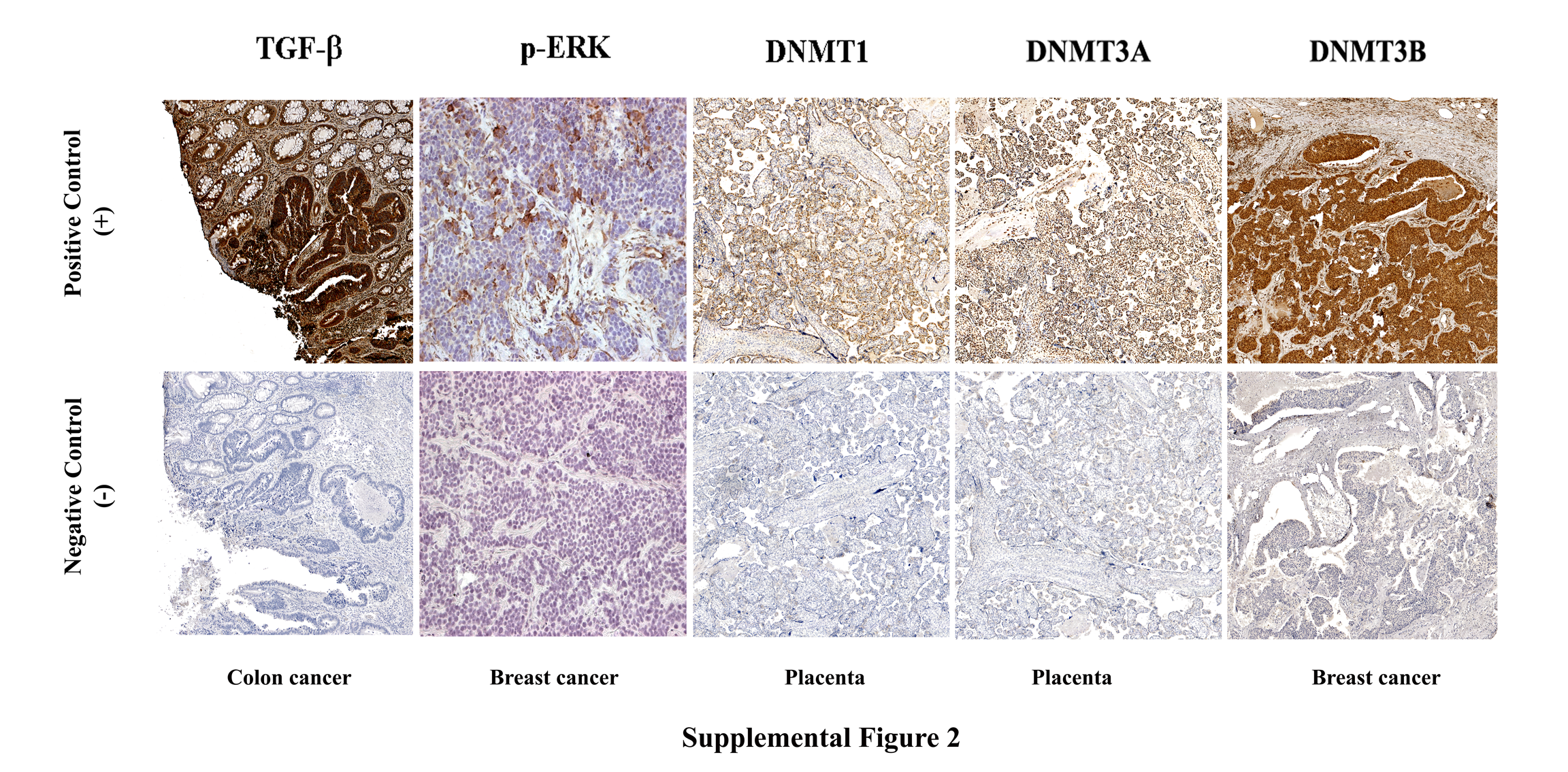

Supplement: Figure S2 — For positive control staining for TMA staining, a tissue (Colon cancer for TGF-β, Breast cancer for p-ERK, Placenta for DNMT1 and DNMT3A, Breast cancer for DNMT3B respectively) which is well known to have expression of target protein was used. Negative controls were identical array sections stained in the absence of primary antibody. (TIF) [file pone.0025168.s002.tif]

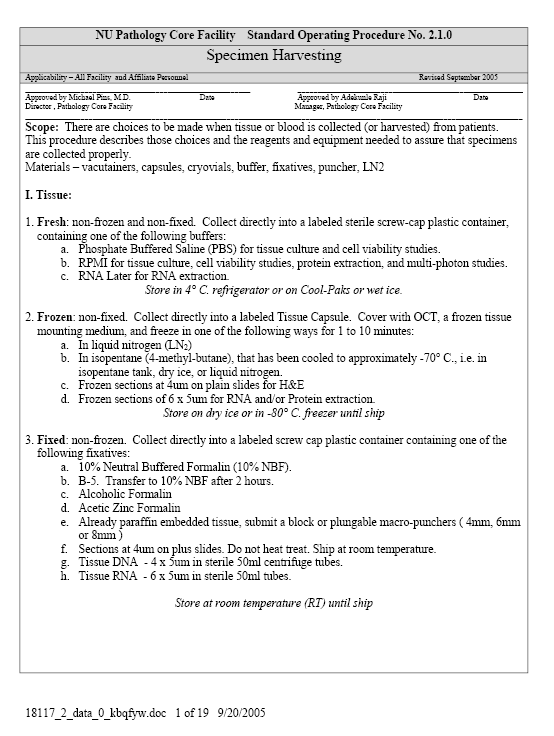


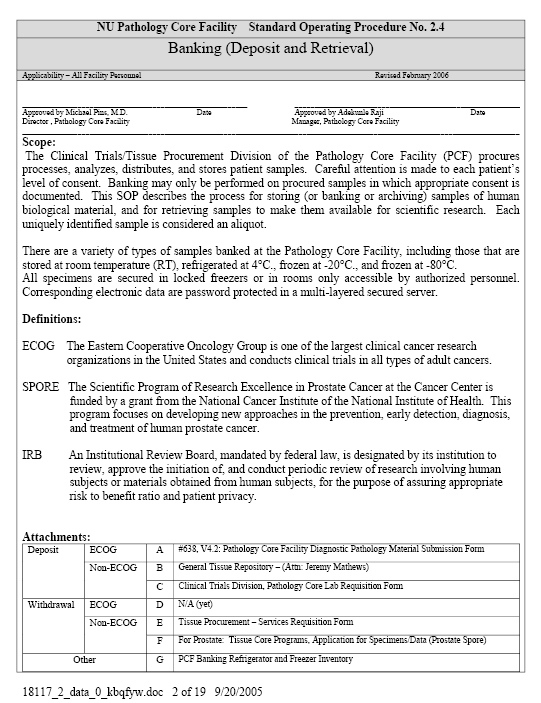


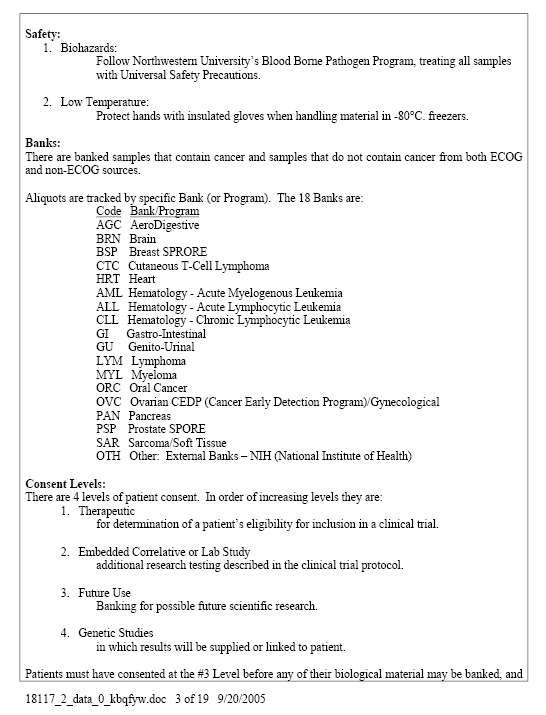


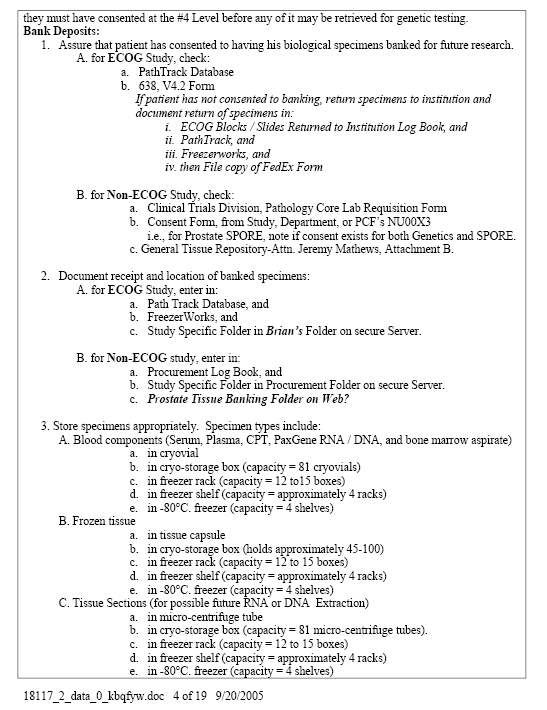


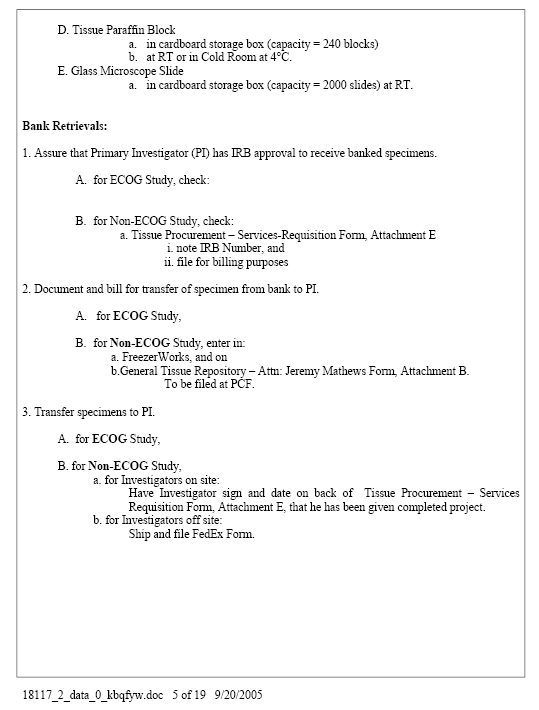


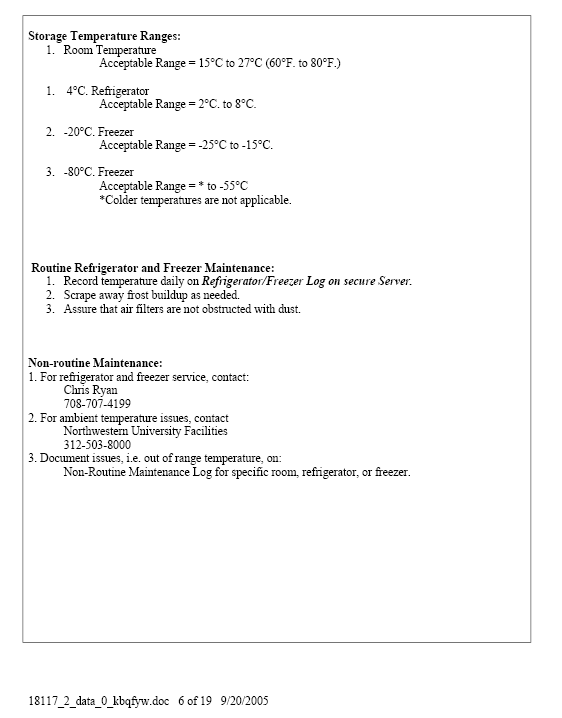


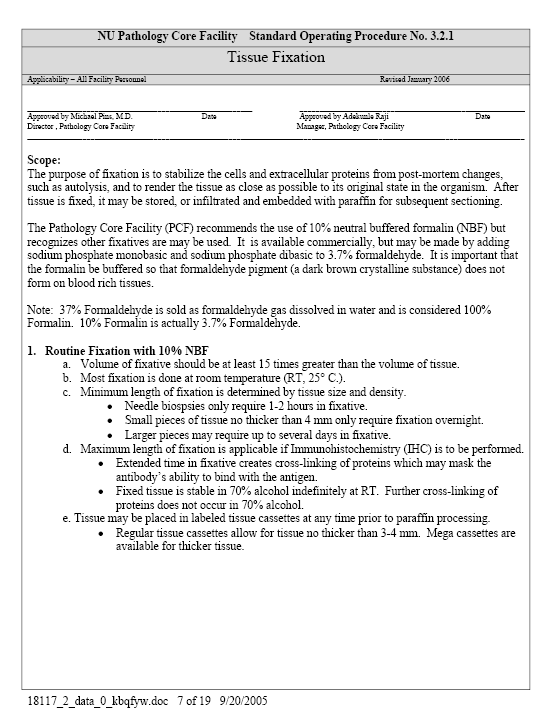


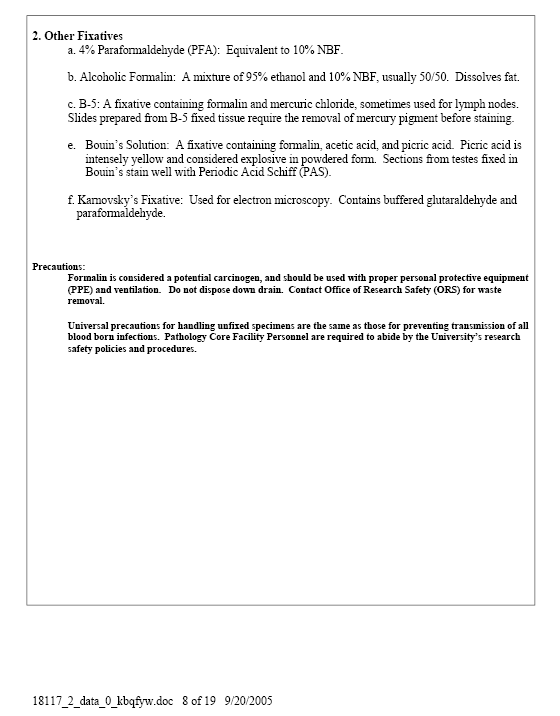


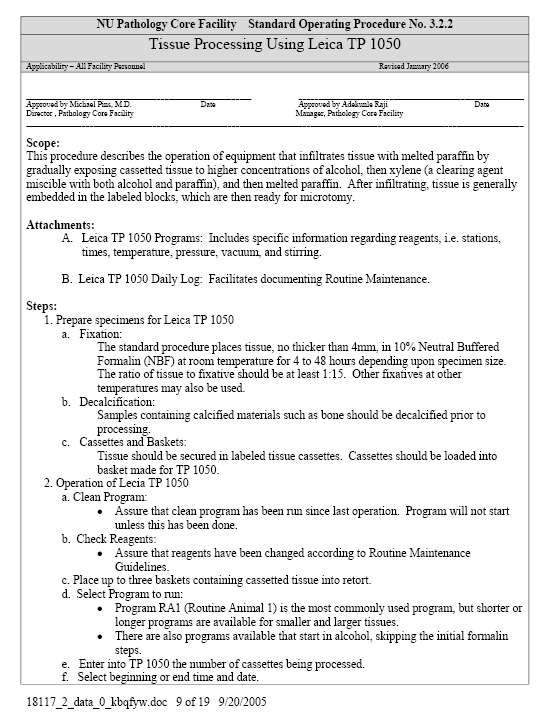


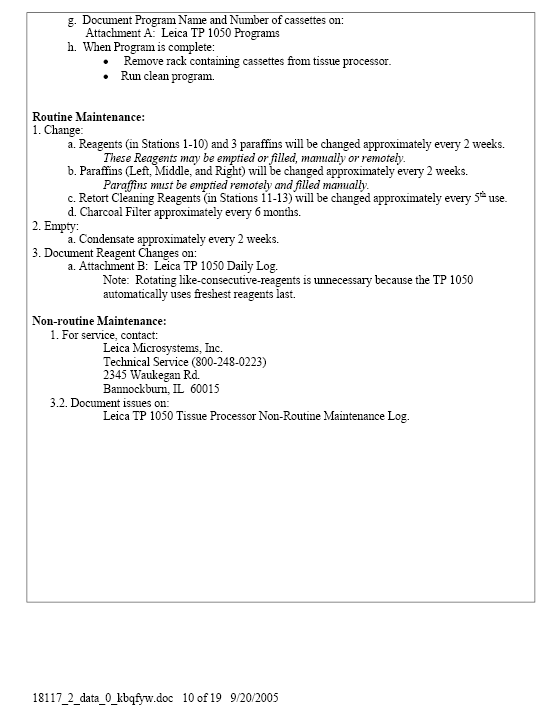


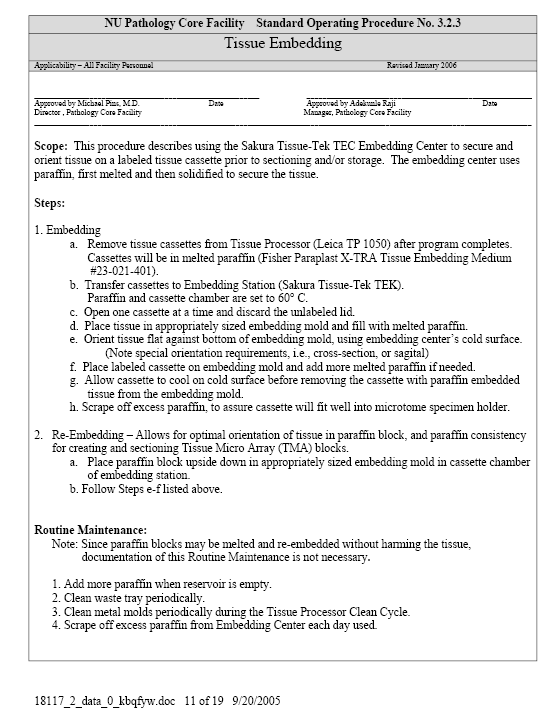


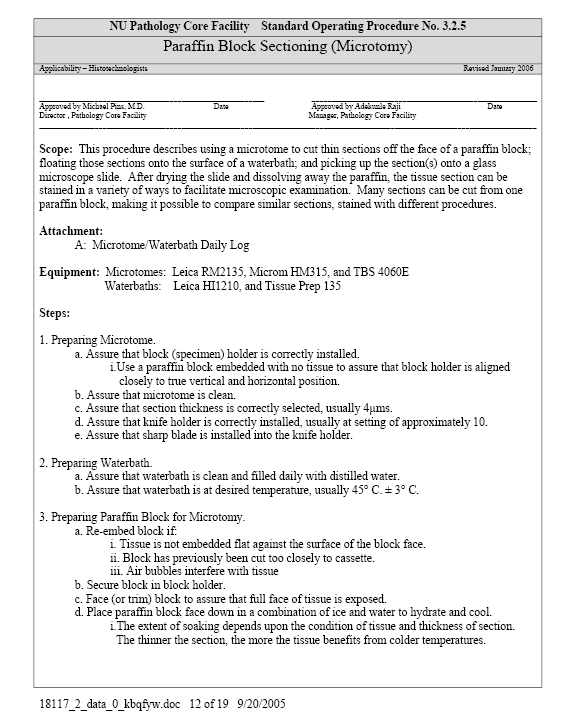


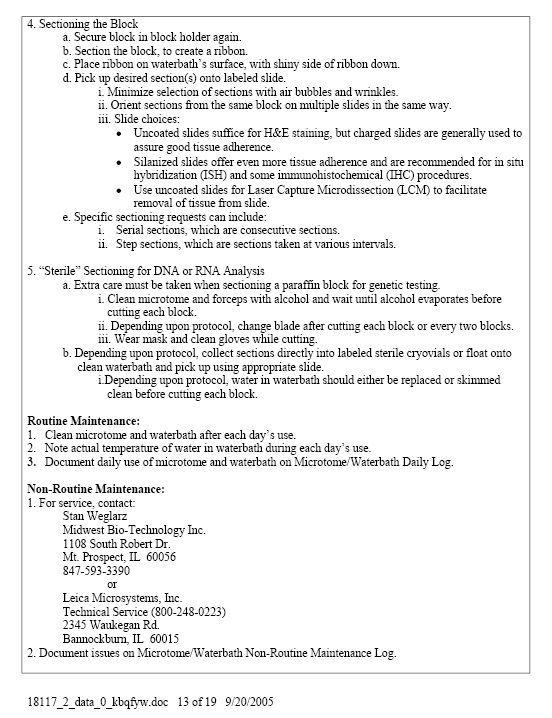


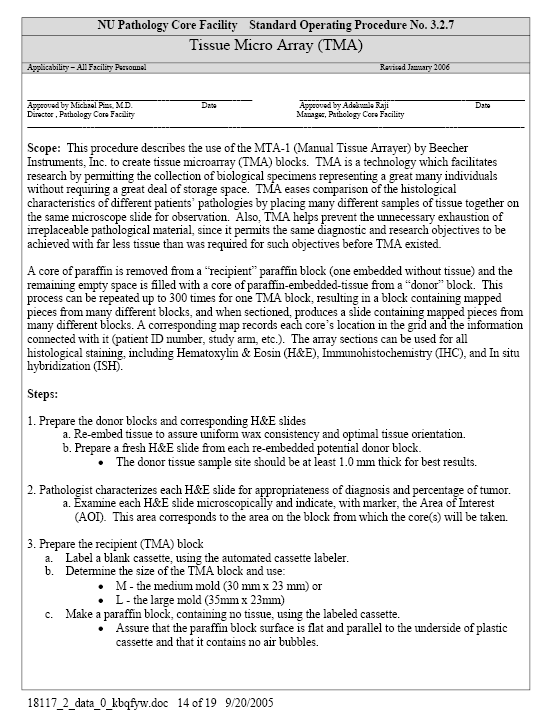


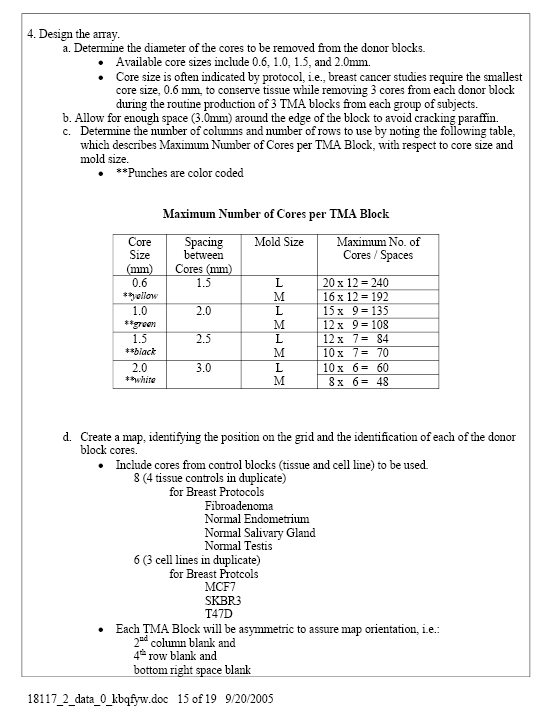


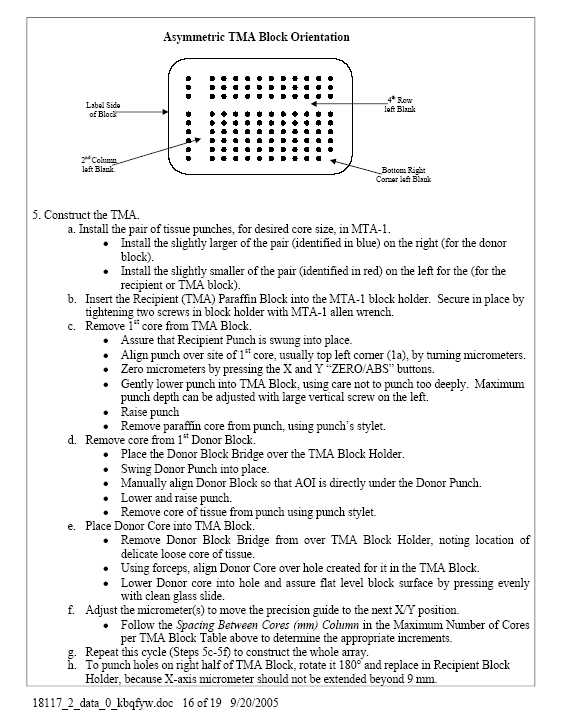


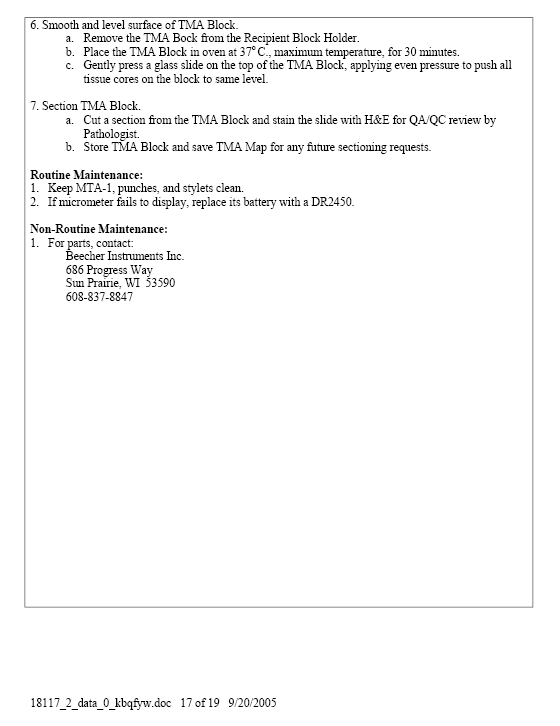


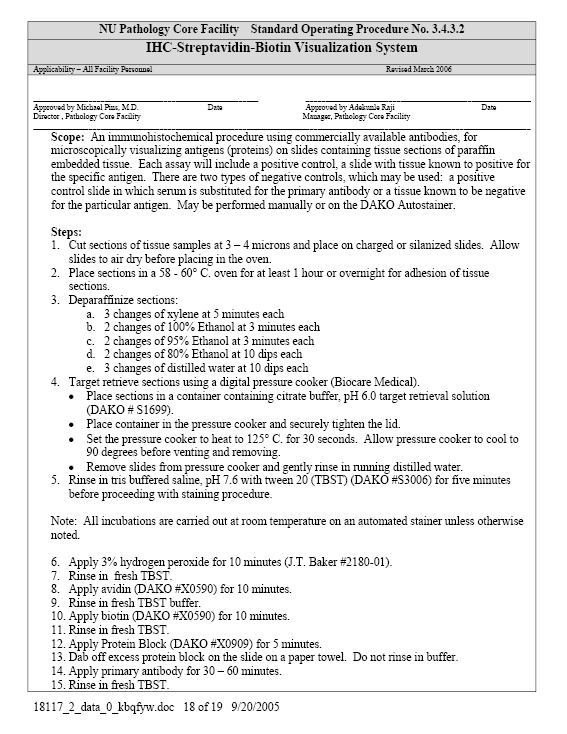


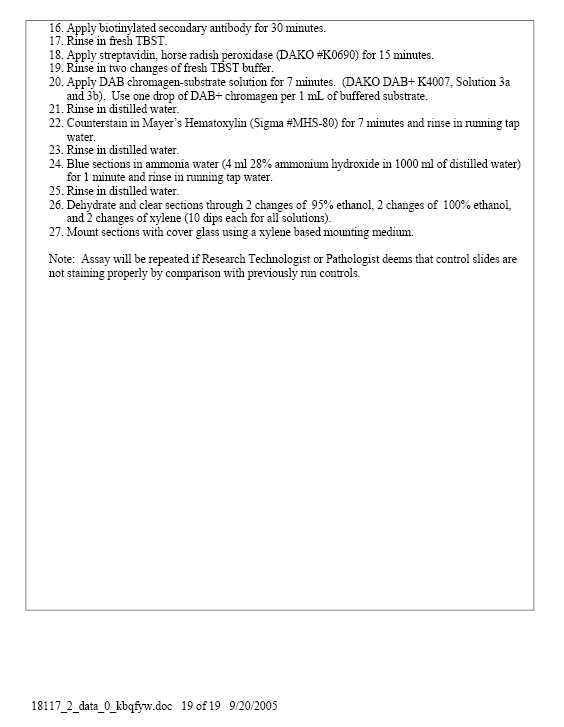

Supplement: Method S2 — NU Pathology Core Facility Standard Operating Procedure. (DOC) [file pone.0025168.s004.doc]

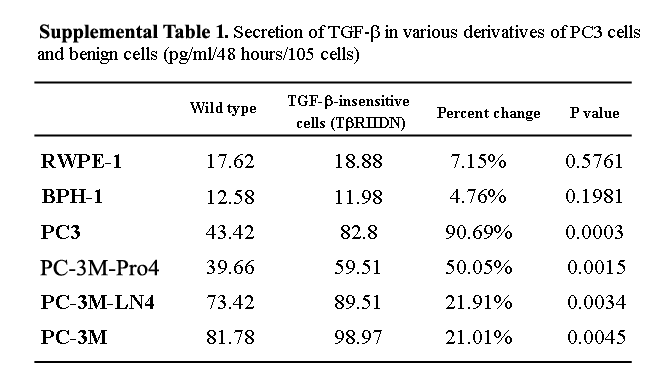

Supplement: Table S1 — Secretion of TGF-β in various derivatives of PC3 cells and benign cells (pg/ml/48 hours/105 cells). (DOC) [file pone.0025168.s005.doc]

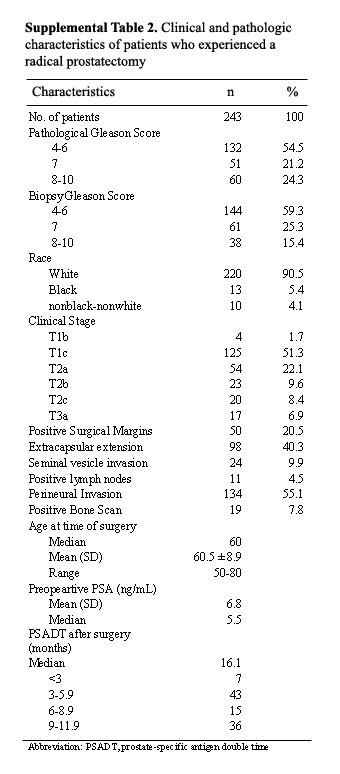

Supplement: Table S2 — Clinical and pathologic characteristics of patients who experienced a radical prostatectomy. (DOC) [file pone.0025168.s006.doc]

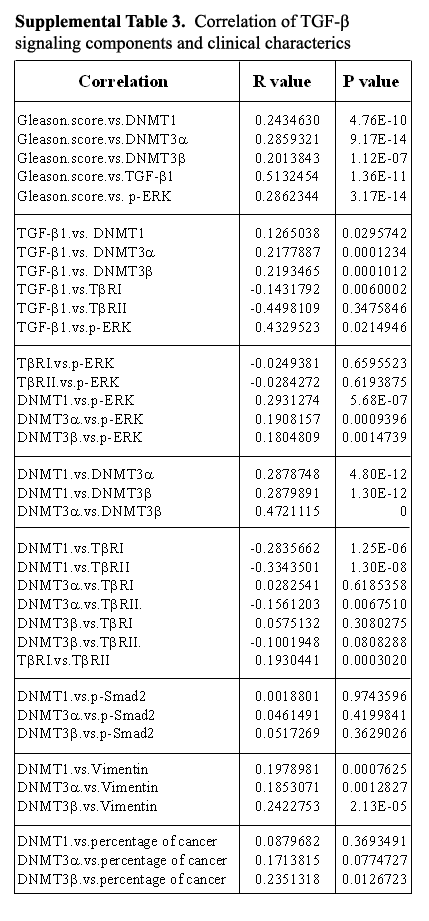

Supplement: Table S3 — Correlation of TGF-β signaling components and clinical characterics. (DOC) [file pone.0025168.s007.doc]

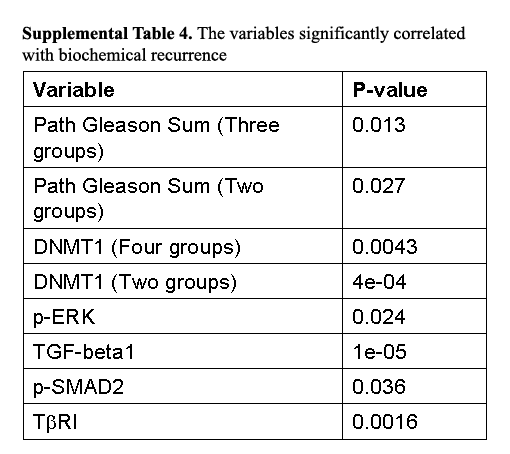

Supplement: Table S4 — The variables significantly correlated with biochemical recurrence. (DOC) [file pone.0025168.s008.doc]

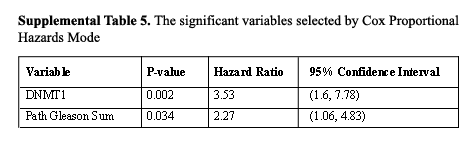

Supplement: Table S5 — The significant variables selected by Cox Proportional Hazards Mode. (DOC) [file pone.0025168.s009.doc]
