# Supplementary material for: TGF-β Regulates DNA Methyltransferase Expression in Prostate Cancer, Correlates with Aggressive Capabilities, and Predicts Disease Recurrence
Source: PLoS One. 2011 Sep 30;6(9):e25168. doi: 10.1371/journal.pone.0025168 (PMC3184137; doi:10.1371/journal.pone.0025168)
Supplement: Method S1 — Supplemental Materials and Methods. (DOC) [file pone.0025168.s003.doc]

**Supplemental Materials and Methods**

***Cell Lines.*** For some experiments, the mouse prostate cancer cell line TRAMP-C2 cells (obtained from Dr. N. Greenberg, Fred Hutchinson Cancer Research Center, Seattle, WA) were grown and maintained in RPMI-1640 containing 10% heat-inactivated fetal bovine serum (FBS, GIBCO), as previously described (21). The benign human prostate epithelial cell line, RWPE-1, was purchased from American type culture collection (ATCC). BPH-1 cells were kindly provided by Dr. Simon Hayward of Vanderbilt University. Four variants of the human prostate cancer PC-3 cell lines were kindly provided by Dr. Fidler and Dr. Pettaway of the MD Anderson Cancer Center (17)**.** These variants included PC-3 (wild type), PC-3M-Pro4 (selected for cancer cells confined to the prostate), PC-3M (selected for metastasis) and PC-3M-LN4 (selected for metastasis to lymph nodes). The androgen insensitive, TGF-β sensitive PC-3 cells were originally established by Ed Kaighn from bony metastasis. PC-3M cells were selected for metastasis by Kozlowski and Fidler (18-20), and later, Dr. Fidler’s laboratory generated various derivatives including PC-3M-LN4 and PC-3M-Pro4 cells (17). PC-3M-LN4 was selected for their capacity to undergo metastasis; while PC-3M-Pro4 was selected for those remained in the primary tumors. It has previously been demonstrated that the PC-3 and PC-3M-Pro4 are less aggressive than PC-3M and PC-3M-LN4 cell lines (17, 22). All PC-3 cells and benign cell lines were cultured in RPMI-1640 medium (GIBCO) supplemented with 10% FBS (GIBCO) (Cells were not tested recently because they have been using for several years). For some experiments, cells were rendered insensitive to TGF-β (as a negative control) by introducing a dominant negative TGF-β type II receptor vector (TβRIIDN) as previously described (21). The infection efficiency was above 95.9% for the TβRIIDN vector in all PC3 cell lines, and blockade of TGF-β signaling was confirmed by lack of p-Smad2 signaling as described before (27). In some experiments, cells were treated with or without TGF-β1 (10ng/ml/24 hours, R&D), and in other experiments cells were treated with TGF-β1 at 10ng/ml or MEK inhibitor U0126 (Promega) at 5μM. Finally, some experiments involved the use of Anti-TGF-β (1, -2, -3) neutralizing mAb (clone 1D11; a gift from Genzyme Corporation) as previously described (23, 24).

***TGF-β1 ELISA.*** RWPE-1, BPH-1 and all PC3 variants and the corresponding TβRIIDN infected cell lines were cultured in fresh serum-free media for 24 hours (1.0 x 10^7^ per T75 flask). The pooled conditioned medium was collected and concentrated by using YM-3 Centriprep Centrifugal Filter Devices (Millipore). TGF-β1 ELISA was carried out using the Quantikine Human TGF-β1 Immunoassay Kit (R&D Systems). The total number of cells in each flask was counted using a Coulter Counter and levels of TGF-β1 were reported as pg/10^5^ cells/48 hours.

***[^3^H]-Thymidine Incorporation Assay.*** All cells were grown in culture for 48 hours. Cells were then exposed to a medium containing [^3^H]-thymidine (0.5 µCi/mL; Amersham Biosciences) for an additional 5 hours. The experiment was terminated by washing with warm serum-free medium and then NaOH (0.1 mol/L) was added to all cell culture wells (1 mL). An aliquot of 100 µL was removed for measurement of the protein content and the remainder was used for the determination of the radioactivity as in counts per minute (CPM). Thymidine incorporation was expressed as the fraction of counts found in cells of untreated controls.

***Western blot analysis.*** Western blot analyses were performed to compare TβRs, DNMTs and ERK expression after different treatments over time. Cell lysates were prepared by adding lysis buffer (50 mmol/L Tris-HCl, 1% NP40, 0.25% Na-deoxycholate, 150 mmol/L NaCl, 1 mmol/L EDTA, 1 mmol/L Na_3_VO_4_, and 1 mmol/L NaF) to cell pellets. An aliquot of approximately 30 µg of total protein was loaded onto 10% acrylamide gel in Tris-HCl (Bio-Rad). Electrophoresis was carried out in Tris-glycine-SDS running buffer and transferred to a polyvinylidene difluoride membrane. Blots were immunoprobed for DNMT1 (ABCAM) at a concentration of 1:500, DNMT3α (Santa Cruz) at a concentration of 1:500, DNMT3β (ABGENT) at a concentration of 1:500, phosphorylated ERK1/2 (p-ERK1/2, Cell Signaling Technology) at a concentration of 1:1000, ERK1/2 (Cell Signaling Technology) at a concentration of 1:1000 and TβRI (Santa Cruz) or TβRII (Cell signaling Technology) at a concentration of 1:500, and they were then stripped and re-probed for glyceraldehyde-3-phosphate dehydrogenase (GAPDH, Advanced Immunochemical) with a monoclonal antibody at a concentration of 1:300. Proteins of interest were detected with the enhanced chemiluminescence detection kit (Amersham Biosciences) followed by exposure to Kodak X-OMAT AR film.

***Methylation-Specific PCR (MSP) and Sequencing*.** Bisulfite Treatment of Genomic DNA
MSP for the methylation status of TβRs was performed according to our previous report (4). The MSP primers were designed according to the sequence of the TßRI (GenBank accession nos. U51139 and GI 1255263) and TßRII promoters (GenBank accession nos. U37070 and GI 1110498)(4).

***Immunofluorescence and Co-staining.*** Immunofluorescence studies were performed on all PC-3 and PC-3 cell line derivatives as previously described (22, 25). For co-localization of DNMTs and phosphorylated ERK (p-ERK), cells were analyzed by using nucleus (DAPI)-DNMTs(TR)-p-ERK (FITC) triple staining. All slides were de-paraffinized and blocked by normal serum. Stained slides were viewed with Nikon TE2000-U fluorescent microscopy (Nikon Corporation, Tokyo, Japan). Images were digitized by Photoshop 7.0 with a PC computer.

***Cell invasion assay.*** Cell invasion assay (Matrigel invasion assay) was done in a 24-well Transwell chamber (8 μm pore size; CytoSelect; Cell Biolabs). Cells were plated at a density of 0.5 × 10^6^ to 1.0 × 10^6^/mL in serum-free medium. TGF-β1 and/or Erk inhibitor UO126, 5’-Aza were added directly to the cell suspension, and 24 h later, the suspension was aspirated and the invaded cells were counted with a light microscope under high magnification objective (×100; Olympus) and measured at A560 nm in a plate reader after treatment with the extraction solution.

***Quantitative RT-PCR.*** Human benign prostate epithelial cells RWPE-1 and BPH-1, and prostate cancer cell lines PC-3 serials) were cultured in fresh media for 24 hours, then exposed for 24 hours to either: 1) external recombinant TGF-β1 (10ng/ml), 2) anti-TGF-β neutralizing monoclonal Ab (1D11; 5μg/ml), or 3) MEK inhibitor U0126 (5μM). Total RNA was extracted using an RNeasy kit (Qiagen). Primers for human for DNMT1, DNMT3α, and DNMT3β (26) were listed as following: DNMT1, 5'-GGAAGGCTACCTGGCTAAAGTCAAG-3' (sense) and 5'-ACTGAAAGGGTGTCACTGTCCGAC-3' (antisense); DNMT3α, 5'-TGGAGAATGGCTGCTGTGTGAC-3' (sense) and 5'-CACTCATCCCGTTTCCGTTTG-3' (antisense); and DNMT3β, 5'-AGTGACCAGTCCTCAGACACGAAG-3' (sense) and 5'-ATCAGAGCCATTCCCATCATCTAC-3' (antisense). Primers for GAPDH are 5'-CATGTTCGTCATGGGTGTGAACCA-3' (sense) and 5'-AGTGATGGCATGGACTGTGGTCAT-3' (antisense). Real-time PCR was performed using iQ SYBR Green Supermix (Bio-Rad Laboratories) in triplicate.

***Animal Studies.*** This study received approval from the institutional review board of Northwestern University (Evanston, IL, Approval protocol number 2007-0565, Supplemental Letter 1). An expression vector encoding a triple fusion protein, containing HSV1-tk-GFP-luciferase (SFG-nTGL) was obtained from Dr. Andy Minn (University of Chicago) (27) with permission from Dr. Vladimir Ponomarev (Memorial Sloan Kettering Cancer Center) (28). Mouse prostate cancer TRAMP-C2 cells were transfected with this reporter gene expression vector, designated as TRAMP-luciferase. This approach permitted for direct monitoring of tumor load in intact animals and the effects of therapy through a non-invasive isoluminescence (IVIS) imaging technology. The study was initiated using the approach of subcutaneous (sc) injection of TRAMP-luciferase cells (5×10^6^ cells) into the right flank region of 30 C57BL/6 mice as described earlier (25). Animals were randomly assigned to three groups following an intraperitoneal injection with the specific anti-TGF-β neutralizing antibody 1D11 or control antibody 13C4 [clone 13C4; a gift from Genzyme Corporation] was used as previously described (23, 24). The experimental groups are described as follows: Group 1: mice received 1D11 at a loading dose of 100 mg/kg intraperitoneally on the day of the injection. Subsequently, Group1 received 1D11 at a dose of 50 mg/kg, respectively, every 3 days until the end of the experimental period; Group 2 mice received the isotype control antibody, 13C4, at an initial loading dose of 100 mg/kg intraperitoneally on the day of the injection and 50 mg/kg in subsequent injections, with an interval of every 3 days; and Group 3: received no treatment as a control. Tumor growth was monitored in real-time using the IVIS 100 imaging system (Xengen Corporation). All the mice were sacrificed after 15 injections of antibodies and group 3 were sacrificed on the same time interval. The size and weight of tumor, weight of prostate and spleen were measured. Tumors were isolated, fixed with formalin and cut into 4-μm sections for hematoxylin-eosin, p-ERK and DNMTs immunostaining (see below).

***Construction of Tissue Microarrays (TMAs) and Clinical Outcome Assement.*** The existing clinical case information and banked tissue established within our prostate cancer Specialized Programs of Research Excellence (SPORE) program database at Northwestern University was used. A series of prostate tissue microarrays (TMA) were constructed with formalin-fixed, paraffin-embedded radical prostatectomy specimens as described previously (4, Supplemental Method 2). At least three cores were taken from each donor tissue block. A total of 243 radical prostatectomy specimens were available with associated clinical information. The pathologist assigns a grade to the most common [tumor](http://en.wikipedia.org/wiki/Tumor) pattern, and a second grade to the next most common tumor pattern. Then, the two grades are added together to get a Gleason Score. For example, if the most common tumor pattern was grade 4, and the next most common tumor pattern was grade 3, the Gleason Score would be 4+3 = 7. The Gleason Score is also known as the Gleason Sum. These specimens included 132 cases with low Gleason grade (≤6), 51 cases with intermediate Gleason grade (=7; 3+4 and 4+3), and 60 cases with high Gleason grade (≥8) (Supplemental Table 2). None of the included patients underwent adjuvant therapy following radical prostatectomy. A retrospective analysis of outcome assessment was performed based on anonymous clinical information linked to the TMA specimens. Biochemical recurrence was defined as a post-operative rise in serum PSA greater than 0.2 ng/ml. The median follow-up period, defined as the time to documented PSA recurrence or to last contact in non-recurring patients, was 60 months (range 22−82). All enrolled subjects provided written informed consent by Northwestern Memorial Hospital and the study was approved by the Northwestern University Institutional Review Board (The IRB number is 1480-002, Supplemental Letter 2).

***Immunohistochemistry.*** All antibodies were first tested and optimized on whole-tissue sections and test arrays as previously described (4, Supplemental Method 2). For positive control staining, a tissue (Colon cancer for TGF-β, Placenta for DNMT1 and DNMT3A, Breast cancer for DNMT3B respectively) which is well known to have expression of target protein was used. Negative controls were identical array sections stained in the absence of primary antibody. Once an appropriate dilution was determined, a set of three slides containing all patient samples were stained for each antibody, using standard two-step indirect immunohistochemistry. After deparaffinization with xylenes, the TMA sections were rehydrated using graded alcohol solutions. Endogenous peroxidase was quenched with 3% hydrogen peroxide in methanol at room temperature (25 °C). The sections were placed in a 95°C solution of 0.01 M sodium citrate buffer pH 6.0 for antigen retrieval. Normal goat serum (5%) was applied for 30 min to block non-specific protein-binding sites. Primary mouse monoclonal antibodies were applied for 30 min at room temperature at the following dilutions: DNMT1 at 1:100 (ABCAM), DNMT3α at 1:100 (Santa Cruz), DNMT3β (ABGENT) at 1:100, [TGF-](http://www.biocompare.com/natureproducts/go.asp?id=nature03672_p_p2)β1 at 1:200 (DAKO), phosphorylated ERK (p-ERK) at 1:200 (Cell Signaling Technology), total ERK (t-ERK) at 1:500 (Cell Signaling Technology), phosphorylated Smad2 at 1:500 (Santa Cruz), TβRI (TGF-β type I receptor; Santa Cruz) at 1:100, TβRII (TGF-β type II receptor; Cell Signaling Technology) at 1:200. For positive control staining, a tissue (Colon cancer for TGF-β, Breast cancer for p-ERK, Placenta for DNMT1 and DNMT3A, Breast cancer for DNMT3B respectively) which is well known to have expression of target protein was used. Negative controls were identical array sections stained in the absence of primary antibody. Following secondary antibody exposure (need details of secondary) all sections were exposed to diaminobenzidine (DAB). Sections were counterstained with Harris's haematoxylin followed by dehydration and mounting. Immunostaining was determined using the Dako [Envision System](http://www.biocompare.com/natureproducts/go.asp?id=nature03672_p_p7). Semiquantitative assessment of all TMAs was performed by 2 independent viewers (Q.Z and X.J.Y.), who were blinded to all clinical information. The frequency of nuclear positive target cells (range 0−100%) in prostatic glandular epithelium was scored for each TMA core as previously described (4). Results of immunohistochemical staining were expressed as the percentage of cancer cells that stained positive for a given antibody and were scored on a scale of 0 to 3: Scale 0 (less than 20% cell staining), Scale 1 (20-49% cell staining), Scale 2 (50-74% cell staining) and Scale 3 (75-100% cell staining) (29, 30).

***Statistical Analysis.*** Numerical data were expressed as mean ± SD. ANOVA and multiple range tests were performed to determine differences of means among different treatment groups. *P* < 0.05 was considered statistically significant. The SPSS 10.0.7 software package (SPSS, Inc.) was used for all analyses. Kaplan-Meier survival curve was analyzed by the log-rank test using the Graphpad Prism 4.02 software (Graphpad Software). To investigate an association with biochemical recurrence, we first used the log rank test to determine whether various markers showed any significant effect on PSA recurrence. We tested variables including all TMA markers, and TGF-β1, DNMTs, p-Erk, pathologic Gleason grade, clinical stage, surgical margin status, patient age, extracapsular extension, seminal vesicle invasion, positive lymph nodes, perineural Invasion and positive Bone Scan. Age in years was stratified as follows: <60, 60-69 and ≥70. TMA pathologic Gleason grade was averaged across all the TMAs and divided into three groups: 4-6, 7 and 8-10. The concentration of all protein markers were divided according to the percentage of staining as scored on a scale of 0-3 (see above). Cox Proportional Hazards Model was fit to include all the significant variables and backward selection method was used to eliminate non-significant variables.

ABBREVIATIONS:

TR Texas red

FITC fluorescein isothiocyanate

GAPDH glyceraldehyde-3-phosphate dehydrogenase

PSA prostate-specific antigen

ELISA enzyme-linked immunoassay
