# Supplementary material for: TGF-β Regulates DNA Methyltransferase Expression in Prostate Cancer, Correlates with Aggressive Capabilities, and Predicts Disease Recurrence
Source: PLoS One. 2011 Sep 30;6(9):e25168. doi: 10.1371/journal.pone.0025168 (PMC3184137; doi:10.1371/journal.pone.0025168)
Supplement: Letter S1 — Northwestern University ACUC Approval protocol number 2007-0565. (PDF) [file pone.0025168.s010.pdf]

Office for the Protection of Research Subjects

Animal Care and Use Committee

Northwestern University
750 N Lake Shore Drive
Suite 700
Chicago, Illinois 60611

acuc@northwestern.edu
Phone 312-503-9339
Fax 312-503-0112

NORTHWESTERN UNIVERSITY

August 8, 2007

Chung Lee
Department of Urology
Tarry 16-733
Chicago Campus

Protocol: 2007-0565
TGF-beta Antibody in Pre-clinical Cancer Immunotherapy

Approved Animal Numbers/Species: 265 Mice
Funding Agency: Genzyme Corporation

The Northwestern University Animal Care and Use Committee (NUACUC) approved the use of animals on the ASP referenced above for the three year period 8/8/2007 through 8/7/2010.

Northwestern University has an Animal Welfare Assurance on file with the Office of Laboratory Animal Welfare (**A3283-01**). Northwestern University conducts its reviews in accordance with United States Public Health Service (USPHS) regulations and applicable federal and local laws. The composition of the NUACUC meets the requirements of the USPHS policy and the Animal Welfare Act Regulations.

For the Committee,

CJ Heckman

CJ Heckman, Ph.D.
Chairman, NUACUC

/hl

cc: OSR
CCM
