# Supplementary material for: TGF-β Regulates DNA Methyltransferase Expression in Prostate Cancer, Correlates with Aggressive Capabilities, and Predicts Disease Recurrence
Source: PLoS One. 2011 Sep 30;6(9):e25168. doi: 10.1371/journal.pone.0025168 (PMC3184137; doi:10.1371/journal.pone.0025168)
Supplement: Letter S2 — Approval letter by the Northwestern University Institutional Review Board (The IRB number is 1480-002). (PDF) [file pone.0025168.s011.pdf]

IRB Review - Office Use Only	IRB Date Stamp - Office Use Only	IRB Accession Number
Northwestern University Institutional Review Board IRB #: 1480-002 APPROVED: 04/29/2008	RECEIVED APR 25 2008 OPRS	200804-2751 Office Use Only
		IRB Project Number: **1480-002**

Northwestern University – Office for the Protection of Research Subjects
REVISION SUBMISSION FORM

☐ **Please check if this is in response to a pending issue**
Name of IRB Staff who requested the change: ____

Please do not submit responses to pending issues on this form unless it has been requested by our office. Responses can be submitted electronically or in paper as a memo along with a cover memo to the person requesting the change.

For ease of submission, please submit your revisions electronically:

- Scan the documents (in PDF format), and e-mail them to irb@northwestern.edu.
- Use this format for the subject line of your message:
  " Protocol Revision [IRB#]"

You can also send or bring them to the appropriate OPRS office:

- Biomedical: OPRS, Rubloff, 7th Floor, 750 N. Lake Shore Drive, Chicago, IL 60611
- Social/Behavioral: OPRS, Hogan, G100-6th Floor, 2205 Tech Drive, Evanston, IL 60208.

HANDWRITTEN FORMS WILL NOT BE ACCEPTED.

1. Submission Date: 4/25/2008
2. Principal Investigator Name: Qiang Zhang, PhD
Phone:**8-8145** Fax:**8-7275** E-Mail : q-zhang2@northwestern.edu
3. Submission Prepared By:
Phone: **8-1998** Fax: 8-7275 E-Mail:**e-kinney@northwestern.edu**
4. Current Project Title: Losee of Expression of TGF-Beta Receptors Triggers the Progression of Human Prostate Cancer

5. Type of Protocol
☒ **Biomedical** ☐ **Social/Behavior Science**

6. Revision Description:
This new revision was initiated by: ☒ Investigator ☐ Study Sponsor
Please check all applicable categories:
☐ Change in Authorized Research Personnel (please attach an updated personnel sheet found on our web-site)
☐ Revised Consent/Consent and Authorization for Research Form (Please specify the type of submission)
☒ Change in Protocol/Procedures (this includes inclusion/exclusion criteria, data collection, and recruitment)*
☐ Protocol Amendment # dated
☐ Revised Investigator's Brochure Version Date:
Does this new IB represent any changes to risks listed in the current approved consent form? ☐ YES ☐ NO
☐ New/Revised Subject Recruitment Materials (Please specify if this is new or a revision of previously approved material in Section 7).
Please check all applicable categories:
☐Newspaper/ Printed Periodical ☐Internet1 ☐Brochure ☐Poster ☐TV/Radio
☐Letter to a potential participant ☐Scripts (for verbal contacts)2 ☐Physician Letter
☐Videotapes (only script needed) ☐Other (Explain)

[1] Provide a copy of the printed version
[2] Submit scripts for all verbal contacts (including what may be verbally discussed with media over the telephone)

☐ Change in total number of subjects to be consented or cases/persons/records to be studied
☐ Change in Risks: Attach new version of consent Version Date:<br>☐ Change in Principal Investigator**<br>New Principal Investigator's Signature: ____________________ Date: ____________________<br>☒ Change in Title:* (Please indicate the new Protocol title in Section 7)<br>☐ Change in study site(s)*<br>☐ Change in HIPAA Compliance*<br>☒ Change in funding source(s)*<br>☐ Other:* Please specify:<br>**Note: Revisions that may affect your HIPAA forms are marked with a *.**

7. **Description of Changes**

Instructions:

- Refer to the Revision Guidelines on the IRB website for additional information.
- Fill in the current status of each item to be revised in the left column below.
- Fill in the proposed revisions in the right column. Include the rationale for each revision. When the protocol has been revised, provide page numbers for each revision.
- **To enable a prompt and accurate review, all changes to the protocol, consents, HIPAA documents or recruitment materials must be highlighted. Revisions without highlighted changes will be returned to the investigator without review.**
- Version dates on all materials should be updated accordingly.
- If additional space is required, attach an additional sheet.

Both Columns below must be completed.

PRESENT SITUATION	REVISION REQUESTED
IRB 1480-002 Loss of the Expression of TGF-Beta Receptors Triggers the Progression of Human Prostate Cancer was funded by the American Cancer Society: Grant Number: ACS-IRG-93-037-12. There was collection of patient data for survival analysis.	We are requesting to change the title to, "Prevent Recurrence by PSMA-Reactive TGF-Beta Insensitive Memory T Cells funded by the ACS, grant number 08-22. We will not collect any patient data, we will be using surgical specimens from operations which are housed in Pathcore. All specimens are de-identified.

VA ACOS Research and Development Name and Signature (If applicable) Date

[signature] 4/25/08

Investigator's Name and Signature Date

Please check all applicable sites listed below to be copied on this submission:

- ☐ AIDS
- ☐ CRO
- ☐ GCRC
- ☐ NCCR
- ☐ RIC
- ☐ NMH
- ☐ VA

☐ TRANSPLANT
☐ Other:

X-Original-To: mcc516@lulu.it.northwestern.edu
Delivered-To: mcc516@lulu.it.northwestern.edu
X-Originating-Ip: 165.124.232.25
Organization: Northwestern University
Priority: 3 (Normal)
X-Webmail-User: qzh758@localhost
To: m-cardona@northwestern.edu
Cc: e-kinney@northwestern.edu
X-Http_host: hecky.it.northwestern.edu
From: Qiang Zhang <q-zhang2@northwestern.edu>
Subject: Re: IRB# 1480-002 revision
Date: Fri, 02 May 2008 9:58:20 -0500
X-Mailer: EMUmail 5.2.7 (UA Mozilla/4.0 (compatible; MSIE 6.0; Windows NT 5.1; SV1))
Reply-To: Qiang Zhang <q-zhang2@northwestern.edu>

Dear officer,

I am sorry for the confusion. The correct title is the first one that "Prevent Cancer Recurrence by PSMA-Reactive TGF-B-Insensitive Memory T Cells."
Thank you so much for the reminder!

Best regards,

Qiang Zhang, M.D.& Ph.D.
Research Assistant Professor
Department of Urology, Northwestern University
303E, Chicago Avenue, Tarry 16-726
Chicago, IL, 60611
Tel: 312-908-7963
Fax: 312-908-7275

==============Original message text===============
On Fri, 02 May 2008 9:50:01 am CDT Carolyn Cardona wrote:

We received a revision submission with a change of title for IRB# 1480-002.

The copy of the grant has the title as "Prevent Cancer Recurrence by Psma-Reactive TGF-B-Insensitive Memory T Cells.

The revision submission page 2 has the change of title to be: Prevent Recurrence by PSMA-Reactive TGF-Beta Insensitive Memory T Cells.

The highlighted words are the ones that are different in the titles. Please clarify which is the correct title to be used. ===========End of original message text===========

X-Original-To: mcc516@lulu.it.northwestern.edu
Delivered-To: mcc516@lulu.it.northwestern.edu
From: "Erin Kinney" <e-kinney@northwestern.edu>
To: "'Carolyn Cardona'" <m-cardona@northwestern.edu>,
<q-zhang2@northwestern.edu>
Subject: RE: IRB# 1480-002 revision
Date: Fri, 2 May 2008 09:56:16 -0500
X-Mailer: Microsoft Office Outlook 12.0
Thread-Index: AcisY3ey3nLonOoNR8uP8h2174khfQAAORnw

Carolyn,

Thank you for the email. I apologize. The revision submission page 2 should include the word "Cancer" in the title, while the short hand for Beta is B.

Therefore, please use the title of the grant, "Prevent Cancer Recurrence by PSMA-Reactive TGF-B-Insensitive Memory T cells.

Please let me know if we may furnish anything further.

Thank you,
Erin

~~~~~~~~~~~~~~~~~~~~~~~~~~~~~~~~~~~~~~

Erin E. Kinney
Northwestern University

Phone: (312) 908 - 1998
Fax: (312) 908 - 7275
Email: e-kinney@northwestern.edu

From: Carolyn Cardona [mailto:m-cardona@northwestern.edu]
Sent: Friday, May 02, 2008 9:50 AM
To: q-zhang2@northwestern.edu
Cc: e-kinney@northwestern.edu
Subject: IRB# 1480-002 revision

We received a revision submission with a change of title for IRB# 1480-002.

The copy of the grant has the title as "Prevent **Cancer** Recurrence by Psma-Reactive TGF-**B**-Insensitive Memory T Cells.

The revision submission page 2 has the change of title to be: Prevent Recurrence by PSMA-Reactive TGF-Beta Insensitive Memory T Cells.

The highlighted words are the ones that are different in the titles. Please clarify which is the correct title to be used.
~~~~~~~~~~~~~~~~~~~~~~~~~~~~~~~~~~~~~~
